# Interpretable prediction of DNA replication origins in *S. cerevisiae* using DNABERT and DNABERT-2

**DOI:** 10.1186/s12859-026-06562-5

**Published:** 2026-07-22

**Authors:** Zohreh Piroozeh, Ildem Akerman, Olga V. Kalinina, Stefan Kesselheim, Alina Bazarova

**Affiliations:** 1 Jülich Supercomputing Centre, Forschungszentrum Jülich, Jülich, Germany; 2 Centre for Bioinformatics, Saarland University, Saarbrücken, Germany; 3 Institute of Biomedical Research, University of Birmingham, Birmingham, UK; 4 Helmholtz Institute for Pharmaceutical Research Saarland (HIPS), Helmholtz Centre for Infection Research (HZI), Saarbrücken, Germany; 5 Medical Faculty, Saarland University, Homburg, Germany; 6 Helmholtz AI, Munich, Germany; 7 Faculty of Mathetmatics and Natural Sciences, University of Cologne, Cologne, Germany

**Keywords:** DNA replication origins, Budding yeast, DNABERT, XAI, Tokenization

## Abstract

**Background:**

DNA replication is a biological process in which a single DNA molecule is duplicated, initiating from multiple genomic sites known as replication origins. Identifying replication origins and analyzing their underlying base sequence composition is crucial for understanding the mechanisms of DNA replication. Although there are various machine learning and deep learning approaches for origin prediction, many rely on labor intensive feature engineering or lack interpretability. We fine-tune two genome-based pretrained language models, DNABERT and DNABERT-2, to predict replication origins in budding yeast and unravel the DNA base composition behind them. The key contribution of this study is a systematic framework for analyzing genomic language models for replication origin prediction, combining controlled dataset design with model-specific explainability pipelines to examine how different tokenization strategies influence learned sequence features and whether such approaches can highlight biologically meaningful signals.

**Results:**

We evaluate both models on the designed datasets to ensure robustness and support explainability. DNABERT demonstrates consistent performance, achieving an average accuracy of 0.72 for more challenging and 0.83 for the easier dataset. In comparison, DNABERT-2 achieved comparable scores of 0.72 and 0.81 on the same datasets. Our attention-based motif discovery pipeline enhances the interpretability of DNABERT, by identifying motifs from high-attention fragments that closely match known sequence patterns of replication origins. Perturbation-based explanation methods, including Shapley additive explanations, were applied to interpret DNABERT-2’s learning mechanism. This analysis identified tokens with high attribution scores aligned with biologically relevant sequence composition.

**Conclusion:**

Our study demonstrates that both models identify replication origin sequences, albeit through different learning strategies. Tokenization appears to influence model learning and attention behavior in these models. The overlapping k-mer tokenization used in DNABERT yields more interpretable attention maps compared to the byte pair encoding tokenization employed in DNABERT-2. We show that despite sharing the same BERT-style architecture, DNABERT captures relevant short-range patterns and some sequence dependencies beyond just local context, as reflected in its attention maps. In contrast, DNABERT-2’s alternative tokenization strategy biases its learning toward relevant short-range patterns by optimizing token weighting.

**Supplementary material:**

The online version of this article (10.1186/s12859-026-06562-5) contains supplementary material, which is available to authorized users.

## Background

For all living organisms, DNA replication is a fundamental biological process that produces two copies from a single DNA molecule, ensuring precise transmission of genetic information during cell division and across generations. Understanding this process is vital, as disruptions in DNA replication can lead to genomic instability, resulting in cellular dysfunction and disease [1]. While prokaryotic cells typically rely on a single origin of replication, eukaryotic cells initiate DNA replication from multiple sites along their chromosomes, known as replication origins (ORIs). This allows replication to initiate simultaneously at several sites, significantly accelerating the overall process. Identifying ORIs in eukaryotes and their sequence features is essential to unravel the underlying mechanisms that govern DNA replication.

Although the motif sequences behind the replication origins remain unclear in most eukaryotes, the search for the replication origins in the *S. cerevisiae* genome (budding yeast) led to the discovery of autonomous replication sequences (ARS). ARS regions are about 100–200 base pairs in length and consist of several key elements: A, B1, B2 and, in some cases, B3 elements [2]. The A element contains the ARS consensus sequence (ACS), together with B1, which forms the main binding site for the origin recognition complex (ORC). The B2 region contains a sequence similar to the ACS and is thought to potentially serve as a secondary ORC binding site, or as a docking site for the core components of the replicative helicase.

The ACS motif was originally characterized by Broach et al. [3], as an 11-base pair T-rich sequence, WTTTATRTTTW,^1^ which was later refined to WTTTAYRTTTW [4]. Subsequently, Theis and Newlon [5] proposed an extended 17-bp version of the motif, WWW-WTTTAYRTTTW-GTT, representing a longer and more complete form of the ACS.

While the ACS is functionally crucial, it is not sufficient on its own to define active origins, as over 12,000 ACS motif matches exist in the budding yeast genome, but only around 500 correspond to functional replication origins under normal conditions [6]. This indicates that there are likely other mechanisms at play, including transcriptional activity that can disrupt origin function [7, 8], chromatin structure, which helps recruit the ORC [9, 10], and secondary sequence motifs that provide additional signals [11, 12].

Several machine learning approaches, such as support vector machines (SVM) and random forests, have been proposed to identify ORI. These methods typically rely on intensive feature engineering. For example, Chen et al. [13] leveraged the structural properties of DNA to represent sequences, while other studies encoded sequences as vectors that capture the nucleotide composition, information on sequence order, and some additional physicochemical properties of DNA [14–17]. Singh et al. [18] proposed a multi-view ensemble learning (MEL) approach to identify DNA replication origins in yeast, using diverse subsets of features based on sequence composition, physical and structural properties. Each subset trained a separate classifier, and predictions were combined via weighted averaging. Among the algorithms tested, SVM achieved the best performance for the prediction of ARS.

Do and Le [19] applied XGBoost [20] with a hybrid feature set combining biological and sequence-derived properties, providing higher accuracy than several earlier methods [13, 14, 17], but still requiring extensive feature selection. Similarly, yORIpred [21] integrated multiple classifiers with diverse feature encoding schemes, yielding high accuracy but depending heavily on manual intervention, high-dimensional feature engineering, and iterative optimization. Wu et al. [22] proposed a deep learning framework that combines Word2Vec-based sequence embeddings with a convolutional neural network (CNN) to identify DNA replication origins. Their approach requires explicit sequence preprocessing and feature construction before model training, after which the resulting embedding matrices are used as inputs to a CNN classifier. The method was evaluated on four datasets. Although the model achieved a reported accuracy of 0.97 on the *S. cerevisiae* dataset, outperforming several earlier approaches, the framework requires training the deep learning model from scratch, which can be computationally expensive, particularly for large genomic datasets. Moreover, the integration of deep learning with embedding-based representations can limit interpretability, as the learned features are implicit and not directly associated with predefined biological descriptors.

In contrast, transformer-based models offer high performance and maintain interpretability, yielding insights into the learning machinery and feature importance. This makes them especially well-suited for tasks involving sequential patterns, such as decoding nucleic acid sequences.

In this study, we investigate whether genomic language models can recover biologically meaningful sequence signals associated with replication origins using large language models (LLMs) such as DNABERT [23] and DNABERT-2 [24]. The well-characterized replication origin system of *S. cerevisiae*, where key sequence determinants such as the ACS motif are known, provides an ideal benchmark for evaluating model interpretability. Using this benchmark, we assess whether pretrained DNA language models recover established biological signals rather than spurious sequence patterns. In addition, we perform an exploratory analysis on human replication origin data to examine whether the proposed interpretability framework can reveal biologically meaningful sequence signatures in organisms where origin determinants are less clearly defined.

Since the main focus of our study is the explainability of the results, we designed a set of diverse datasets with varying levels of complexity, incorporating non-origin subsampling through different strategies. This setup enabled us to compare model performance and analyze how each model behaves when challenged to discriminate between classes with distinct properties across these datasets. Therefore, both models were fine-tuned on the two biologically motivated datasets to predict replication origins in budding yeast. Additional shuffled datasets were used as controlled evaluation settings to further assess model behavior under sequence perturbations. To enhance the interpretability of DNABERT, a comprehensive pipeline was developed to identify the underlying sequence motifs using attention maps. We also discussed the explainability of the attention maps extracted from the fine-tuned DNABERT-2, as well as its learning mechanism, using various interpretability approaches, resulting in the identification of the tokens receiving consistently high attribution scores in the model’s predictions.

Importantly, our objective was not to optimize raw accuracy on a single dataset or establish performance superiority between architectures, but rather to evaluate whether pre-trained DNA language models can reduce the need for extensive manual feature engineering, avoid computationally expensive training from scratch, and provide interpretable insights through attention- and SHAP-based analyses.

Although other DNA language models are available [25, 26], we initially chose DNABERT because of its straightforward process to extract attention maps, commonly used as an explainability tool to highlight the most relevant input components and meaningful correlations that explain the model’s predictions. We subsequently extended our analysis to DNABERT-2, a particularly relevant model for comparison, though its attention maps differed substantially and were more difficult to interpret. To overcome this challenge, we employed alternative explainability approaches, including perturbation-based methods.

## Materials and methods

### Datasets

Among the available data sources for DNA replication origins of the budding yeast genome, OriDB [6] provides a highly curated collection of experimentally confirmed origins, making it an ideal reference for cross-study comparisons. We considered 325 of confirmed ORIs, none longer than 500 bp, as positive instances to meet DNABERT’s input size requirement. Shorter ORIs were asymmetrically extended with real genomic flanking nucleotides of the corresponding origin sequence, to obtain sequences of uniform length (500 bp). The required extension length was randomly allocated between the left and right sides of the ORIs, while ensuring that the extended regions did not exceed the boundaries of the chromosome or overlap with neighboring annotated ORI. This random allocation allows the ORI to occur at different positions within the 500 bp window and avoids introducing systematic positional bias that the model could otherwise exploit during classification. Although this extension may result in additional noise for shorter origins, it standardizes the input length and frames the task as distinguishing origin-containing sequences from non-origin genomic regions, which better reflects the real-world biological challenge of identifying functional replication origin regions against a genomic background. In addition, because our explainability pipeline is attention-based, we examined whether the model assigns higher attention scores to the true ORI region compared to the extended flanking sequences.

In genome analysis studies, replication origins constitute only a small portion of the genome, making generation of non-origin samples challenging and crucial for model performance. In our previous efforts to identify ORIs using DeepGRN [27], we generated positive and negative origin instances through a sliding-window approach across the entire genome, assigning class labels based on their overlap with annotated ORIs. However, this strategy resulted in a low true-positive rate, primarily due to pronounced class imbalance. This imbalance resulted in a high overall validation accuracy ($\sim 0.94$) but poor performance on the positive class, with precision below 0.2 and recall around 0.02.

To overcome this limitation, we assembled two balanced datasets of different difficulty levels, each tailored to investigate specific research questions about the base composition of ORIs and model learning behaviour. Our data engineering strategy involves subsampling diverse non-origin sequences to create two datasets, each containing the same set of positive instances (replication origin sequences) but different sets of negative instances representing non-origin sequences. The datasets are described as follows:

- **Random-Neg dataset**: To create 325 non-origin sequences, negative instances were randomly chosen from parts of the genome that did not overlap with any origin sequences. This approach, commonly employed in related studies (e.g., [19, 22]), ensures a diverse representation of non-origin sequences derived from real genomic data. The dataset is available in Additional File 2.
- **ACS-Neg dataset**: We aimed to identify discriminative features beyond ACS motifs by subsampling negative instances containing at least one non-replicating ACS motif. Initially, we considered positive instances that have at least one ACS match, resulting in 298 out of the total 325 origin sequences in our primary dataset being selected. Then, the same number of negative instances were subsampled from approximately 12,000 ACS matches identified by HOMER [28], where the motif matrix was generated based on the representation of the ACS motif with 17 bp, WWW-WTTTAYRTTTW-GTT [5]. The ACS matches were selected and extended to 500 bp in such a way that they did not overlap with ORIs (positive instances). ACS motif match strength was assessed using the HOMER MotifScore, which reflects the log-likelihood similarity between a sequence and the ACS position weight matrix. In the ACS-Neg dataset, motif strength was closely matched between positive and negative sequences (mean MotifScore 11.7 vs 11.5), with no statistically significant difference (Mann–Whitney U-test $p = 0.387$; Kolmogorov–Smirnov test $p = 0.514$). In contrast, negative sequences in the Random-Neg dataset exhibited substantially weaker ACS matches (mean MotifScore 6.6; Mann–Whitney U-test $p < 10^{-43}$). Therefore, the ACS-Neg dataset provides a more stringent and controlled setting for evaluating ACS motif–based discrimination, as it removes motif strength as a distinguishing feature between classes. The dataset is available in Additional File 3.

This approach allows for a comparison of model performance across datasets with distinct negative-sampling strategies, offering deeper insights into the discriminative features of replication origin sequences that influence model predictions.

### Methods

DNABERT is a genome-based large language model (LLM) adapted from the original BERT architecture, the transformer encoder, and pre-trained on human genome using overlapping k-mer tokenization. The BERT architecture consists of a stack of Transformer encoder layers with multi-head self-attention and position-wise feed-forward networks that produce contextualized representations of input tokens [29]. DNABERT contains approximately 86 million parameters, which is fewer than the standard BERT-Base (110 M) due to a smaller vocabulary size and reduced embedding matrix. DNABERT-2 follows the same architecture as its predecessor DNABERT, maintaining the same number of layers (12) and hidden dimensions (768), but is pre-trained on multi-species genome data, including human, mouse, yeast, and virus sequences. In addition to expanded training data, DNABERT-2 incorporates architectural optimizations such as Attention with Linear Biases (ALiBi) for length extrapolation and Flash Attention for computational efficiency. Furthermore, DNABERT-2 adopts an alternative tokenization strategy, replacing overlapping k-mer tokenization with byte pair encoding (BPE) [30]. BPE generates variable-length tokens by iteratively merging the most frequent contiguous sequence segments in the dataset to build the vocabulary. For DNABERT-2, a vocabulary of 4,096 tokens was constructed and used for sequence tokenization. This configuration results in a model containing approximately 117 million parameters. Critically, the parameter increase relative to DNABERT stems almost exclusively from the larger embedding matrix required by BPE, rather than an increase in the model’s depth or width. This approach aims to eliminate the partial information leakage inherent in overlapping k-mer tokenization while also benefiting from the computational efficiency of non-overlapping tokenization [24].

Input sequences were formatted following the standard BERT convention. A [CLS] token was added at the beginning of each sequence and a [SEP] token was used to mark the end of the sequence. When tokenization produced sequences of equal length (yeast DNABERT datasets), padding was not required. In cases where tokenized sequences varied in length (e.g., human DNABERT and yeast DNABERT-2 datasets), shorter sequences were padded with [PAD] tokens to match the fixed input length required by the model.

We used pre-trained DNABERT and DNABERT-2 and fine-tuned them for binary sequence classification. A linear classification head was applied on top of the final hidden representation of the [CLS] token, which serves as a global embedding of the input sequence. The classification head consists of a fully connected layer followed by a softmax activation that outputs the probability of the sequence belonging to the origin or non-origin class. Model parameters were optimized using binary cross-entropy loss, and all model parameters were updated during training (full fine-tuning), following the protocols described in the original DNABERT and DNABERT-2 studies.

DNABERT was fine-tuned following the optimization strategy described in the DNABERT study. Training was performed using the AdamW optimizer with weight decay, with the learning rate linearly warming up to a peak value before decaying toward zero. A learning rate of $2 \times 10^{-4}$, a warm-up proportion of 0.1, and a batch size of 32 were used.

DNABERT-2 was fine-tuned following the training configuration described in the DNABERT-2 study. Optimization was performed using the AdamW optimizer with a weight decay of 0.01 and a batch size of 32. The maximum tokenized sequence length was set to 125 tokens, and a warm-up of 50 steps was applied before the learning rate decayed according to the training schedule. The model was trained with a learning rate of $3 \times 10^{-5}$.

Because the two models use different tokenization strategies and were pretrained under different optimization regimes, we adopted the fine-tuning configurations recommended in their respective original studies rather than enforcing identical hyperparameters. This ensures stable optimization for each architecture while maintaining comparable experimental conditions. Details of the fine-tuning hyperparameters are summarized in Additional File 1, Table S1.

For each dataset presented in in Section Datasets, both DNABERT and DNABERT-2 were fine-tuned and evaluated using the same seven random train/validation/test splits (70/10/20) to ensure comparable evaluation conditions. Models were trained for up to 200 epochs while monitoring validation performance. However, model selection was not based on the final epoch. For each run, the earliest checkpoint achieving the maximum combined validation score (accuracy + AUC) was selected and subsequently evaluated on the independent test set. The average of the seven runs was then reported together with the standard deviation. The experimental design further included cross-dataset evaluation to assess how models trained on different negative sets generalize to datasets with shuffled sequences, along with a suite of explainability techniques outlined in the following.

### Explainability approaches

In this study, we focused on model explanations, as the main objective was to determine whether the models can identify the known properties of replication origin sequences, and if their predictions may be influenced by other factors as well. Among the available approaches to model interpretability, we focused on three: attention-based explanations, perturbation-based explanations, and Shapley additive explanations (SHAP) analysis, as described below.

#### Attention-based explanation

DNABERT’s built-in motif analysis tool, which has proven effective for identifying transcription factor binding sites [23], was less suitable for detecting ORI motifs, possibly because ORI motifs tend to be longer. Although it detected motifs partially similar to ACS or A/T-rich flanking regions, the results lacked robustness in *p*-value and motif length. This limitation likely stems from its reliance on continuous high-attention regions as the initial search space. Since the model does not consistently assign high attention across entire continuous regions, significant portions of motifs may be missed by the algorithm. Furthermore, the original procedure relies primarily on clustering of attention-derived segments and does not incorporate probabilistic alignment strategies, which can limit its ability to capture variable motif instances across sequences. To overcome these issues, we developed an alternative pipeline that combines attention-based localization with probabilistic motif inference using the bioinformatics tool MEME (Multiple Expectation Maximization for Motif Elicitation) [31]. MEME employs an expectation–maximization algorithm to align motif instances across sequences and infer a probabilistic motif model, enabling the detection of motifs even when their instances are variable or only partially captured by attention signals. Since visualization of attention scores for the token [CLS] revealed a recurring pattern, characterized by sharp attention peaks which consistently appeared within the actual range of origin sequences, the strategy was to treat fragments surrounding these peaks as target regions for motif discovery.

**Fig. 1:**
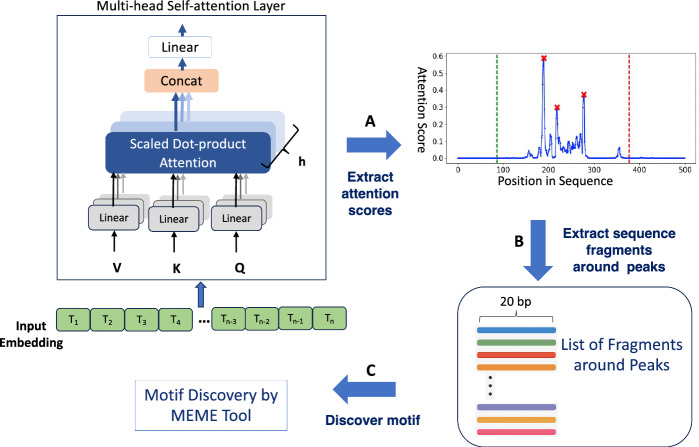
Proposed attention-based motif discovery pipeline. **A** Attention scores for the [CLS] token are extracted from the final hidden layer of the fine-tuned DNABERT model and projected from token resolution to nucleotide resolution (left). The resulting attention scores are visualized along the input sequence (right), where green and red vertical lines indicate the annotated start and end positions of the replication origin. **B** Attention peaks are identified across the sequence to locate regions receiving the highest model attention and sequence fragments (20 bp) centered around highest peaks extracted. **C** Set of selected fragments used as input for motif discovery with the MEME tool

The proposed pipeline, illustrated in Fig. 1, includes three steps as follows: (a) The attention scores for the [CLS] token were extracted from the final hidden layer of the fine-tuned model. To obtain a single vector of attention scores per token and project token-level attention scores to nucleotide resolution, we used the attention score projection strategy introduced by Ji et al. [23] for DNABERT attention visualization. This step is used to derive nucleotide-level attention scores that serve as input to the subsequent steps. (b) High-attention fragments around the peaks were selected. To enhance the robustness of attention-based motif discovery, we restricted the search space to 20 bp fragments surrounding the highest attention peaks. For each input sequence, a limited number of top-scoring peaks (up to four) were selected based on a predefined attention score threshold (0.1), to restrict the motif search to a small subset of each sequence rather than analyzing the full length. In order to limit fragment overlaps, only peaks separated by at least 10 bp were included. (c) A set of selected fragments were processed using MEME motif discovery.

#### Cross-dataset evaluation on shuffled datasets

To further investigate how the composition of the negative training data influences the sequence signals learned by DNABERT, we performed an additional cross-dataset evaluation. Specifically, models fine-tuned on the Random-Neg and ACS-Neg datasets were evaluated, without further training, on perturbed test sets generated from the positive origin sequences. Two types of perturbed negative test sets were constructed:

- **Shuffled-Neg test sets**: negative sequences were generated by randomly shuffling nucleotides within each positive test sequence, thereby preserving overall nucleotide composition while disrupting sequence order.
- **Block-5-Shuffled-Neg test sets**: negative sequences were generated by dividing each positive test sequence into 5 bp blocks and randomly shuffling the order of these blocks. This preserves local sequence structure within blocks while disrupting broader sequence organization.

Because these perturbed negative sequences are derived directly from the positive origin sequences, they preserve much of the compositional properties of the positive class. Evaluating Random-Neg- and ACS-Neg-trained models on these test sets therefore provides a controlled way to assess how strongly the original classification depended on compositional differences between positive and negative training sequences, as opposed to additional sequence-order or origin-associated features learned during fine-tuning.

#### Perturbation-based explanation

Unlike DNABERT, attention maps extracted from fine-tuned DNABERT-2 did not offer sufficient information for explainability. Therefore, we explored alternative interpretation approaches for DNABERT-2.

To explain the performance of DNABERT-2 and investigate the most discriminative sequential features of the model, we leveraged the perturbation-based explanation, which is a type of feature attribution-based explanation [32]. Perturbation-based explanation methods evaluate how the decision-making of a model is influenced by altering or removing parts of the input and observing the effect on the output. The simplest form, leave-one-out, removes or modifies specific input features and measures their importance based on the resulting prediction changes.

To identify the most discriminative features for the model, this approach was applied in two following ways. First, to evaluate the importance of ACS motifs in predictions provided by the fine-tuned DNABERT-2 on Random-Neg dataset, sequences were perturbed by eliminating ACS motifs from the origin sequences. We considered all samples that contain at least one ACS match identified by Homer. By systematically removing these motifs, extending the sequence to its original length and reintroducing the modified samples into the fine-tuned model, we assessed changes in classification probability to evaluate the model’s dependence on ACS motifs.

As an alternative perturbation strategy, each sequence were first tokenized by BPE and the resulting tokens were then randomly shuffled. By feeding these shuffled tokens as a tokenized input into the fine-tuned model, we evaluated how token identity versus token ordering influence the predicted class probabilities. This analysis is particularly relevant given that the DNABERT-2 uses BPE tokenization, which produces non-overlapping tokens.

#### Explaining with Shapley values

For DNABERT-2, insights from both attention map analysis and perturbation experiments, specifically through token shuffling, suggested that the model predictions are strongly influenced by token identity and the presence of specific informative tokens, extending thereby the claim by Sanabria et al. [33]. This observation motivated a systematic analysis of token-level attribution scores to identify tokens receiving consistently high SHAP values in the model’s predictions. To this end, we employed explainable artificial intelligence (AI) techniques using SHAP [34], a perturbation-based method designed to quantify feature-level contributions to model predictions. SHAP provides local additive feature attributions relative to a specified background distribution by approximating the effect of perturbing input features. These attributions therefore characterize how the model assigns importance to input tokens under this approximation, rather than identifying definitive causal determinants of model behavior. However, they can still provide useful insights into model behavior by revealing tokens that consistently receive high attribution scores across repeated runs.

We leveraged TransSHAP [35], which is a variant of SHAP adapted to explain Transformer-based language models, such as BERT, in text classification tasks within Natural Language Processing (NLP). In TransSHAP, the classifier function perturbs word-level representations of the input, tokenizes the perturbed sequences using the BERT tokenizer, and computes predictions for these locally modified instances, which are then passed to the Kernel SHAP explainer to estimate feature contributions.

We adapted the TransSHAP framework for DNA sequence classification by DNABERT-2, developing a modified module that enables SHAP-based explanations of DNA sequences tokenized with BPE. Original TransSHAP has been adapted for BERT’s context-dependent tokens. It constructs baselines from training data, using either realistic replacement tokens or averaged contextual embeddings. This ensures that any perturbations remain linguistically and semantically coherent, effectively simulating a token’s absence without disrupting the sequence flow. While in NLP the focus is on grammar and semantics, in DNA the key considerations are sequence plausibility and motif integrity.

We used the model fine-tuned on the Random-Neg dataset, which contains a broader and more heterogeneous set of non-origin samples than the ACS-Neg dataset and achieved a higher test accuracy of 81%. Since in Random-Neg dataset the negative class consists of randomly sampled from genomic regions, it provides a relatively diverse representation of non-origin sequences compared with the ACS-Neg dataset. To keep perturbations within the model’s learned distribution, we used the complete Random-Neg training set as the SHAP background distribution because it represents the distribution on which the DNABERT-2 was fine-tuned, making it an appropriate reference distribution for perturbation-based attribution analysis. Shapley values were subsequently computed for tokens in the test set to quantify their contributions to the model’s predictions. However, SHAP attributions are inherently defined relative to the selected background distribution. Consequently, genomic patterns that are underrepresented or absent from the Random-Neg training data may also be underrepresented in the SHAP background, potentially influencing attribution scores for such sequences. Therefore, the resulting explanations should be interpreted as describing the model’s predictions for the replication origin discrimination task under the Random-Neg training distribution, rather than as representing universal feature importance across the entire genomic sequence space.

We ran the module 30 times to account for the stochastic nature of SHAP’s subset sampling. This typically reduces variance in the estimated feature attributions, ensuring that the resulting explanations are robust, reproducible, and not artifacts of random sampling. Since our focus is on identifying tokens contributing to model predictions for each class, from each run we extracted the top 50 tokens with the highest SHAP attribution values supporting the predicted class. We then tallied how frequently each token appeared in top-50 across 30 runs and reported the most frequent ones. This allowed us to identify tokens that consistently receive high attribution scores in the model’s classification decisions. In Tables 4 and Additional File 1 Tables S7–S9, positive Shapley values indicate tokens that support the predicted class, whereas negative values correspond to tokens that counteract the prediction by pushing the instance away from the predicted class.

To quantify the strength of origin-associated sequence signals, we defined a normalized AT-index. The index is a composite measure combining (i) global AT fraction, (ii) the frequency of AT-rich motifs informed by the SHAP attribution analysis, (iii) the length of the longest contiguous AT run, and (iv) the frequency of alternating contiguous AT runs. All components were normalized prior to aggregation to ensure comparable contribution to the final score. The exact formulation of the AT-index is provided in Additional File 1, Supplementary Text B, Table S10.

Differences in AT-index values between sequence groups were assessed using the Mann–Whitney U test. Effect sizes were quantified using Cliff’s delta (Additional File 1, Supplementary Text B).

## Results

### Performance analysis

We utilized the DNABERT model pre-trained on overlapping 4-mers and fine-tuned it with our designed datasets independently. Notably, varying the k-mer length did not lead to any significant differences in performance for this downstream task. Therefore, we report the results of the fine-tuned DNABERT model using 4-mer tokenization across all datasets described in section Datasets. The average performance across seven different dataset splits is reported in Table 1.

**Table 1:** Test-set performance of the fine-tuned DNABERT model across the four designed datasets. Results are reported as averages across seven independent train/validation/test splits (70/10/20).

Dataset	Accuracy	AUC	Precision	Recall
Random-Neg	0.83 (0.04)	0.90 (0.04)	0.83 (0.03)	0.83 (0.03)
ACS-Neg	0.72 (0.04)	0.79 (0.04)	0.73 (0.03)	0.72 (0.04)

Metrics are calculated on the corresponding test sets and include accuracy, area under the ROC curve (AUC), precision, and recall. Standard deviations are reported in parentheses

The model demonstrated considerable performance on the Random-Neg dataset, despite the dataset containing diverse negative samples. As expected, the model performance on the ACS-Neg dataset was lower than on the Random-Neg dataset. In this dataset, ACS motifs are present in both origin and non-origin sequences, making the classification task more challenging because the ACS motif is no longer uniquely associated with the positive class. The observed decrease in accuracy is therefore consistent with the ACS pattern contributing to the model’s predictions in the Random-Neg setting, although other sequence differences between the datasets may also play a role. The accuracy of 0.72 on the ACS-Neg dataset indicates that the model can still identify additional sequence features beyond ACS matches to discriminate origin from non-origin sequences.

The same training strategy for data splits was applied to DNABERT-2, and the results are presented in Table 2, showing the average performance of the fine-tuned model on the test set. DNABERT-2 exhibited lower performance compared to its predecessor in the Random-Neg datasets. While DNABERT achieved 0.83 accuracy and 0.90 AUC, DNABERT-2 showed 0.81 accuracy and 0.82 AUC.

On the ACS-Neg dataset, the model achieved an accuracy of 0.72 with a standard deviation (SD) of 0.05, comparable to DNABERT’s accuracy of 0.72 (SD = 0.04), suggesting its ability to capture discriminative features beyond the ACS motif despite reduced accuracy relative to the Random-Neg dataset.

**Table 2:** Test-set performance of the fine-tuned DNABERT-2 model across the four designed datasets. Results are reported as averages across seven independent train/validation/test splits (70/10/20).

Dataset	Accuracy	AUC	Precision	Recall
Random-Neg	0.81 (0.04)	0.82 (0.03)	0.83 (0.04)	0.81 (0.04)
ACS-Neg	0.72 (0.05)	0.70 (0.08)	0.74 (0.05)	0.72 (0.05)

Metrics are calculated on the test sets and include accuracy, area under the ROC curve (AUC), precision, and recall. Standard deviations are reported in parentheses

To assess the reliability of random data splitting and ensure that it did not lead to an overestimation of performance, we also applied chromosome-based data splitting to the Random-Neg dataset. DNABERT exhibited slightly better performance under this scheme compared to random splitting, whereas DNABERT-2 achieved comparable results to those obtained with random splitting. The average performance across seven chromosome-based splits is summarized in Additional File 1, Table S4. These findings indicate that random splitting did not bias the performance of the models.

Validation performance of both models followed the same trends as the test results and was consistently slightly higher across datasets (Additional file 1, Tables S2–S3). This behaviour is expected because model checkpoints were selected based on validation metrics, and the observed differences between validation and test performance remained small.

The corresponding training and validation loss curves are shown in Additional File 1, Figures S1 and S2. Although validation accuracy and AUROC remained relatively stable throughout training, validation loss increased during later epochs, particularly for DNABERT-2. This apparent discrepancy can be explained by the fact that binary cross-entropy loss and discrimination metrics such as accuracy and AUROC quantify different aspects of model behaviour. Whereas accuracy and AUROC primarily reflect the ability to distinguish between classes, binary cross-entropy is additionally sensitive to prediction confidence. Consequently, a small number of increasingly confident misclassifications may substantially increase validation loss even when overall discrimination performance remains stable or even improves. Similar calibration- and confidence-related effects have previously been reported for high-capacity neural networks and limited validation datasets [36, 37]. Importantly, model selection was based on the earliest checkpoint achieving the maximum combined validation score (accuracy + AUC), and the selected checkpoints generalized consistently to the independent test sets, indicating that predictive performance remained stable despite the observed loss behaviour. Additional discussion of the observed validation-loss dynamics and the properties of binary cross-entropy loss is provided in Additional File 1, Supplementary Text A.

Notably, training both models from scratch yielded an average test accuracy of 0.71 on the Random-Neg dataset for DNABERT, and 0.60 for DNABERT-2. We also evaluated our task using the pretrained models without fine-tuning, resulting in an average accuracy of 0.37 and 0.55 for DNABERT and DNABERT-2, respectively, on the same dataset. Models’ performance on the shuffled datasets in this case was close to random with AUC$\approx 0.5$, indicating that the ability to distinguish shuffled from non-shuffled sequences is not directly implied by the pre-training stage but emerges after task-specific fine-tuning. These observations suggest that both pre-training and task-specific adaptation contribute to model performance. However, the improvement from pretrained to fine-tuned models is larger for DNABERT than for DNABERT-2, suggesting that fine-tuning contributes more strongly to DNABERT’s final performance. One possible contributing factor is that the models differ in their pre-training data: DNABERT was pretrained on the human genome, whereas DNABERT-2 was pretrained on multi-species genomic sequences including yeast. As a result, DNABERT may benefit more strongly from task-specific fine-tuning on yeast sequences, while DNABERT-2 may already encode representations partially aligned with this task.

### Explainability of the results

#### DNABERT

**Fig. 2:**
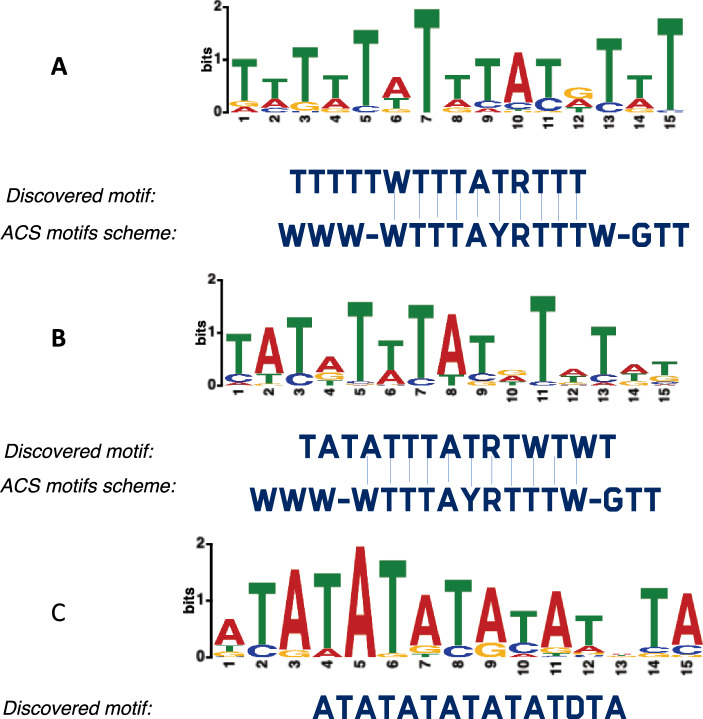
Motifs discovered by MEME from high-attention fragments. Motifs were identified from 20 bp fragments surrounding attention peaks detected in sequences processed by DNABERT. **A** and** B**: Motifs discovered from the test and training sets, respectively, of the model fine-tuned on the Random-Neg dataset,** C**: Motif discovered from the training set of the model fine-tuned on the ACS-Neg dataset. The ACS motif scheme is shown for comparison

For DNABERT, our motif discovery pipeline was applied to the training and test sets of the Random-Neg and ACS-Neg datasets, identifying the most significant motifs. High-attention fragments (20 bps) from TP cases were analyzed using MEME in the classic mode. For the Random-Neg dataset, motifs from the test and train sets are shown in Fig. 2 A, B, as ’TTTTTWTTTATRTTT’ (E-value=2.6e−006) and ’TATATTTATRTWTWT’ (E-value=2.3e−032), respectively. These motifs closely resemble the ACS pattern, specifically the motif from test set shows a strong agreement with the experimentally confirmed ACS pattern 5’-WTTTATRTTTW-3’ [3], underscoring its biological relevance. The difference stems from greater variability in the training set, resulting in broader motif representations than in the test set.

For the ACS-Neg dataset, motif discovery on the test set yielded no significant results, likely due to the small sample size (<45 instances) and worse model performance. However, analysis of the train set revealed the motif ’ATATATATATATDTA’ (E-value=1.6e−026, Fig. 2C), characterized by alternating A/T bases, a pattern associated with the intergenic origins [38].

To assess whether negative predictions were driven by specific motif-like patterns, we applied the same analysis to high-attention fragments from true negative instances in both datasets. No statistically significant motifs were identified, suggesting that negative predictions are not dominated by a single recurrent sequence pattern but instead reflect more distributed sequence features or the absence of strong origin-associated motifs.

**Table 3:** Cross-dataset test-set evaluation of DNABERT models trained on the Random-Neg and ACS-Neg datasets.

Dataset by model	Accuracy	AUC	Precision	Recall
Shuffled-Neg by Random-Neg	0.62 (0.03)	0.69 (0.04)	0.64 (0.03)	0.62 (0.03)
Block-5-Shuffled-Neg by Random-Neg	0.59 (0.02)	0.68 (0.05)	0.61 (0.03)	0.59 (0.02)
Shuffled-Neg by ACS-Neg	0.69 (0.04)	0.81 (0.03)	0.71 (0.04)	0.69 (0.04)
Block-5-Shuffled-Neg by ACS-Neg	0.68 (0.03)	0.81 (0.04)	0.70 (0.03)	0.68 (0.03)

Models were trained on one dataset and evaluated on the Shuffled-Neg and Block-5-Shuffled-Neg datasets to assess generalization across different negative sampling strategies. Metrics are defined as in Table 1

To complement the motif-based analysis and further interpret the sequence signals captured by DNABERT, we additionally performed the cross-dataset evaluation described in Section Cross-dataset evaluation on shuffled datasets. Models trained on the Random-Neg dataset achieved AUC values of 0.68−0.69 on the shuffled datasets, whereas models trained on the ACS-Neg dataset achieved higher AUC values of approximately 0.81, see Table 3. Because the shuffled negatives preserve nucleotide composition of the positive origin sequences, this decrease suggests that compositional differences between origin sequences and randomly sampled genomic negatives contributed substantially to the original classification performance. However, performance remained clearly above the non-fine-tuned baseline ($\sim 0.5$, Section Performance analysis), indicating that the fine-tuned models also captured additional origin-associated sequence signals beyond base composition alone. Notably, performance of the Random-Neg-trained models decreases slightly further on the Block-5-shuffled dataset, where short-range sequence structure is partially preserved in the negative sequences. This suggests that local sequence organization contributes to the learned discriminative signals, thereby increasing similarity between the negative sequences and true origins, consistent with the motifs identified in Figs 2A,B. In contrast, models trained on the ACS-Neg dataset retained similarly high performance on both shuffled test sets, despite the Block-5-shuffled negatives preserving substantially more local sequence structure. Because ACS motif strength was controlled between classes during training, these results, together with the motif identified in Fig. 2C, suggest that ACS-Neg-trained models rely on sequence characteristics distinct from the relatively simple motif- and composition-based signals observed in the Random-Neg setting.

#### DNABERT-2

First, analogously to DNABERT, we investigated the explainability of attention scores for DNABERT-2. However, attention maps extracted from the fine-tuned DNABERT-2 did not display clearly defined or distinctly high-attention regions in the visualizations, as shown in Additional File 1, Fig. S4. Consequently, using attention score visualization to identify potentially important fragments as a target for motif identification proved infeasible. As a result, our proposed motif discovery pipeline was not effective for DNABERT-2. This observation, marked by the limited interpretability of attention maps, aligns with ongoing debates in the explainability literature, which argue that attention maps do not always provide straightforward or clear explanations of model behavior [32].

**Fig. 3:**
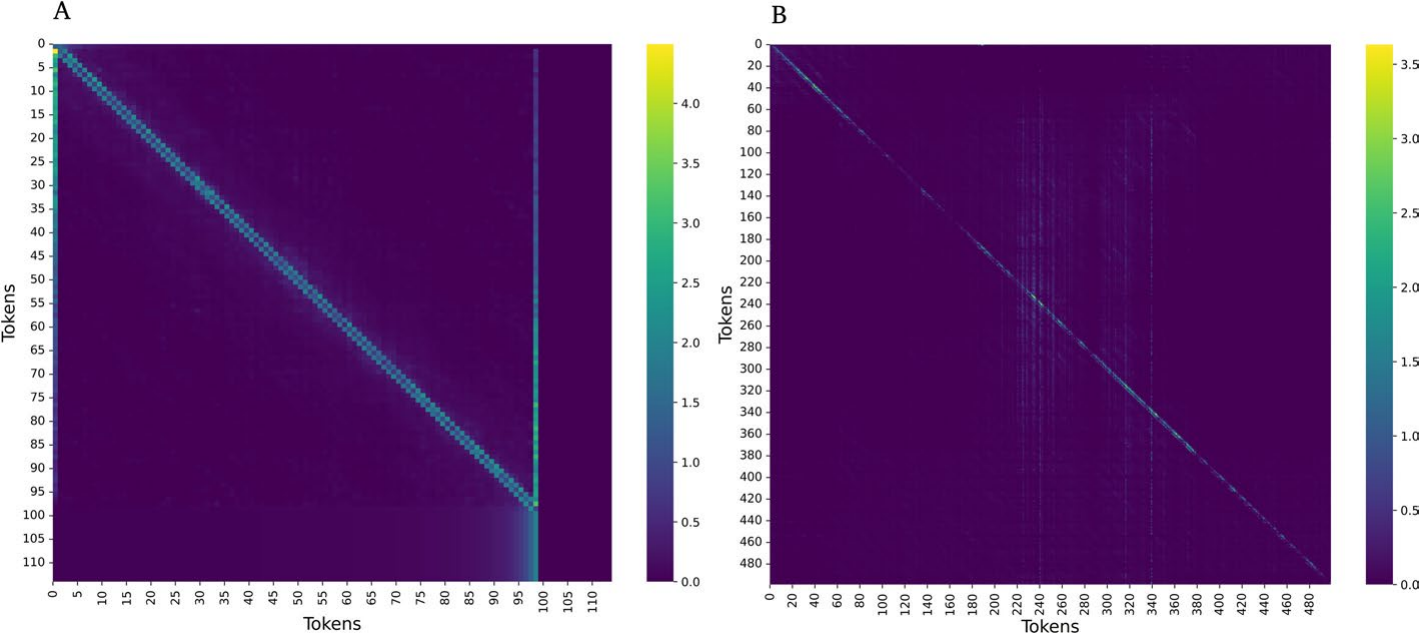
Attention heatmap comparison for an origin sequence correctly classified by both models.** A**: The attention maps obtained from fine-tuned DNABERT-2 reveal strong diagonal patterns, indicating that the model emphasizes short-range dependencies and interactions among neighboring tokens.** B**: In addition to strong diagonal patterns, the attention maps of the fine-tuned DNABERT display non-diagonal high attention regions and partially emphasized columns, suggesting that the model captures long-range sequence dependencies beyond immediate local interactions

However, analyzing attention heatmaps still offers valuable insights into how DNABERT-2 represents tokenized sequences compared to DNABERT. A consistent difference is observed when comparing the attention maps extracted from both models for the same sequences. For example, Fig. 3 shows the attention heatmaps for a specific origin sequence correctly classified by both models. Attention scores are taken from the final layer of each fine-tuned model, with values aggregated across all heads. The distinct attention patterns observed for the same sequence indicate a fundamental difference in how each model captures token relationships, despite being fine-tuned on the same dataset (Random-Neg). DNABERT-2 specifically exhibits strong diagonal attention weights, indicating that the tokens focus mainly on themselves and their immediate neighbors (see Fig.3 A). This suggests that the model processes the input in a more localized manner, relying on short-range patterns rather than capturing global context. Consequently, its predictions appear to be driven by local token-level features, without considerable emphasis on global sequence dependencies. Note that the only prominently highlighted column in the attention heatmap corresponds to the [SEP] token, which marks the end of the sequence. Since BPE produces tokenized sequences of variable lengths from input sequences with same length, and [PAD] tokens are appended to shorter sequences to ensure uniform input dimensions. This explains the attention directed toward the [SEP] position, as it consistently appears near the actual end of meaningful sequence content. In contrast, DNABERT attention maps (see Fig.3 B), in addition to showing some diagonal high attention scores, demonstrate also non-diagonal attention patterns, with certain columns receiving high attention across many positions (i.e., some tokens are attended by many others throughout the sequence). This reflects the model’s capacity to integrate some global sequence dependencies beyond just local context. Moreover, tokens or motifs that are consistently attended to by other tokens can be potentially valuable for motif-based classification tasks, as they may highlight salient sequence regions and reveal informative features for explainability.

To assess the contribution of ACS motifs to DNABERT-2 predictions on the Random-Neg dataset, we perturbed origin sequences by removing ACS motif instances and re-evaluated the modified sequences using the fine-tuned model. We observed that the model’s predictions changed for only 10% of the sequences classified as origins by the model, leading to their reclassification as non-origin sequences. These changes predominantly corresponded to origin sequences that were originally classified as true positives. At the dataset level, this perturbation reduced the overall AUC from 0.82 to 0.77, indicating a measurable but moderate effect on predictive performance. This suggests that, although ACS motifs influence the model’s decision-making process, additional factors also contribute to the predicting ORIs by the model.

As an alternative perturbation approach, we disrupted token order by randomly permuting tokenized sequences and then re-evaluating the shuffled tokens with the fine-tuned model. This allowed us to assess the contribution of token ordering to the predictions. We applied this approach to a model fine-tuned on the Random-Neg dataset, making predictions on the test set for true positive (TP) samples. Notably, after shuffling, all perturbed samples were still correctly classified as positive instances. This suggests that when strong origin-associated sequence signals are present, predictions remain robust even when the original token order is disrupted, relying therefore exclusively on token identity.

In contrast, shuffling had a markedly different effect on negative sequences. The number of TNs decreased by 30% on average across seven shuffling experiments (from 44 to 26), while the number of FPs almost doubled from 21 to 39 on average across seven shuffling runs, thereby decreasing the AUC from 0.82 to 0.75. To investigate the sequence features underlying these predictions, we applied our TransSHAP-based explainability pipeline (Sect. Explaining with Shapley values). The tokens receiving the highest attribution scores across runs are summarized in Table 4. Notably, all highly ranked tokens are enriched in A/T bases. Many correspond to short AT-rich fragments, including patterns with alternating adenine and thymine nucleotides (e.g., TATA-like motifs), which have previously been reported in studies of yeast replication origins and identified by the motif discovery pipeline from Section Attention-based explanation. The recurrence of such tokens across independent runs indicates that the model consistently relies on AT-rich sequence elements when identifying origin sequences.

To further assess the stability of these contributions, we examined the distribution of SHAP values for these tokens across all runs, considering all Shapley values rather than only the top-ranked instances reported in Table 4. For most tokens, the SHAP value distributions were strongly skewed toward positive values, indicating a consistent positive contribution to origin classification. The SHAP value distributions for the top tokens listed in Table 4 are shown in Additional File 1, Fig. S5.

In contrast, tokens supporting negative predictions were considerably more heterogeneous and did not form a single dominant motif class. The complete lists of tokens associated with negative predictions, including those identified in true negatives as well as sequences that remained correctly classified (TN$\rightarrow$TN) or became misclassified (TN$\rightarrow$FP) after token shuffling, are provided in Additional File 1, Tables S7–S9.

To assess whether negatives that became false positives after shuffling contained stronger origin-like sequence signals, we compared the values of AT-index, described in Sect. Explaining with Shapley values across sequence groups. TP sequences exhibited the highest AT-index values (mean = 0.61), whereas correctly classified negatives showed substantially lower values (mean = −0.45). Negatives that became false positives after shuffling displayed intermediate AT-index values (mean = −0.17).

The difference between TP and TN sequences was highly significant with a large effect size (Mann–Whitney $p = 3.4\times 10^{-14}$, Cliff’s $|\delta | = 0.87$), as was the difference between TP and TN$\rightarrow$FP sequences ($p = 2.3\times 10^{-5}$, $|\delta | = 0.73$). In contrast, the difference between TN and TN$\rightarrow$FP sequences was smaller and did not reach statistical significance ($p = 0.14$, $|\delta | = 0.27$), likely reflecting the smaller sample size of this group.

Together, these results suggest that DNABERT-2 predictions are strongly influenced by local AT-rich sequence fragments. When such signals are sufficiently intense, predictions remain stable even after token order is disrupted. However, when the positive signal is weaker, classification appears to depend more on the broader sequence context. Disrupting this context through token shuffling can therefore expose latent origin-like sequence features and lead to false positive predictions.

**Table 4:** Tokens contributing most strongly to origin sequence predictions in DNABERT-2 based on SHAP analysis

Token	Frq. in 30 runs	Total Frq	Avg. Shap values
AAAAAAAA	20	32	0.087552
TAGTTTT	18	29	0.087283
GTGTGTG	15	15	0.070021
TATATATATATATA	13	20	0.078311
TAAAAAAA	12	28	0.069601
CTTAAAT	12	12	0.077911
TATTTT	11	11	0.078129
TATATATATATATATATATATATATATATATA	11	11	0.072893
TATTTAA	11	19	0.069307
AAAAAAAAAAAA	10	10	0.082038
TAAAATTA	10	20	0.076150
TTTTATTT	10	11	0.075111
TATATATA	10	36	0.065474
TGATAAAA	9	9	0.081201
TATAAAA	9	22	0.069639
CTTTTTTT	9	13	0.064365

This table presents the high-impact tokens frequently contributing to class of origin sequences prediction by the fine-tuned DNABERT-2 model. The column **Frq. in 30 runs** indicates the number of SHAP analyses (out of 30) in which a token appeared at least once among the top 50 SHAP-ranked tokens. **Total Frq.** indicates the cumulative number of appearances among the top-ranked tokens, across all runs. **Avg. Shapley values** represents the average of Shapley values across these appearances

### Human data

To examine whether the proposed DNABERT explainability pipeline generalizes beyond *S. cerevisiae*, we applied it to a human replication origin dataset. In particular, we considered the K562 dataset [39], which has been used by machine learning and deep learning approaches for the prediction of human replication origins. Dao et al. [39] proposed iOR-Epi, a machine learning–based method for replication origin prediction using three datasets, including K562 human cell line. Their approach combines sequence-derived features with epigenomic and chromatin interaction data, requiring substantial feature engineering and integration of multiple data types.

More recently, the same benchmark datasets were used in the Ori-FinderH study [40], which applied deep learning DNA, using sequence information alone represented based on the Z-curve [41] transformation. While this approach demonstrated strong predictive performance with AUC = 0.9616 on the human K562 cell line, it still relies on a predefined encoding of nucleotide sequences into engineered numerical features. These studies suggest that, despite the absence of a strict consensus sequence for human replication origins, detectable sequence patterns may still contribute to origin prediction. Motivated by this observation, we applied the DNABERT-based analysis pipeline developed for yeast to the K562 dataset to assess whether biologically meaningful sequence signals can be identified directly from nucleotide sequences using a pre-trained DNA language model for human genome.

#### Dataset

The K562 dataset contains experimentally annotated replication origins (positive samples) together with non-origin genomic regions (negative samples). It is a well-characterized leukemia cell line frequently used in genome-wide replication origin studies. The non-origin regions are sampled from genomic intervals between adjacent replication origins. In total, the dataset includes 62,971 origin sequences and 63,971 non-origin sequences for training and validation, with sequence lengths ranging from 99 to 11,899 bp.

A notable characteristic of this dataset is that origin and non-origin sequences exhibit substantially different length distributions. Specifically, the length distribution of origin samples is restricted to discrete values (e.g., 99 bp, 199 bp, 299 bp, and so on), whereas the lengths of non-origin samples span a much wider range of values. Although DNABERT can process variable-length sequences up to its maximum context length (512 bp), strong systematic differences in sequence length between classes can introduce a potential confounding factor in classification tasks. At the same time, artificially extending shorter origin sequences to a fixed length, as we did with yeast data, could lead to overlaps with neighboring non-origin intervals, thereby introducing unintended intersections between samples, and deviating significantly from the structure of the original dataset.

Therefore, we first curated a dataset based on the original K562 dataset by selecting only sequences shorter than 512 bp; we refer to this subset as K562-L512 dataset, with length distribution same as original K562, shown in Additional File 1 Fig. S6. In addition, we constructed an alternative dataset in which the length distribution of the negative samples was adjusted to match that of the positive samples; this dataset is referred to as K562-LenMatch. Specifically, from the K562 training set we retained all positive sequences shorter than 512 bp and generated the same number of negative samples by randomly sampling contiguous subsequences of matching length from longer negative sequences in K562 so that their length distribution closely followed that of the positive sequences. The resulting training set contains 37,438 positive and 37,438 negative sequences and was split into training and validation sets using an 8:2 ratio for model fine-tuning. An independent evaluation dataset was also available for K562, from which a test set was constructed using the same strategy, with negative sequences matched to the length distribution of the positives. The sequences were extracted using the human genome assembly hg19 following the procedure described in [39]. The length distribution of dataset K562-LenMatch dataset is shown in Additional File 1 Fig. S7, and the number of samples for both datasets is summarized in Additional File 1 Table S5. Because the K562 dataset is substantially larger than the yeast datasets used in this study, a single train/validation/test split was used for this exploratory analysis rather than performing repeated splits.

To assess potential data leakage due to homologous regions and to ensure that random splitting did not lead to overestimation of performance, we additionally performed chromosome-based splitting for both K562-L512 and K562-LenMatch datasets. Specifically, we applied chromosome-level grouped cross-validation, in which entire chromosomes were treated as disjoint groups and assigned to train, validation, and test sets without overlap. This approach enforces strict physical separation within the human genome and mitigates potential leakage arising from sequence homology. We constructed seven such splits and fine-tuned and evaluated the model independently on each held-out test set. The exact chromosome partitions used for these splits are available in the associated code repository, along with the datasets.

#### Performance analysis and explainability

DNABERT was fine-tuned on both datasets using the same hyperparameters as those for budding yeast datasets, but for 7 epochs (hyperparameters are summarized in Additional File 1, Table S1). The validation checkpoint was selected based on the first highest value of validation metrics (AUC+Accuracy) (Additional File 1, Figure S3) and then used for evaluation on the corresponding test set. Results for random data splitting are illustrated in Table 5.

**Table 5:** Test-set performance of DNABERT fine-tuned on two K562 datasets with different length distributions.

Dataset	Accuracy	AUC	Precision	Recall
K562-L512	0.996	0.997	0.996	0.995
K562-LenMatch	0.894	0.956	0.895	0.894

K562-L512 corresponds to the dataset containing sequences shorter than 512 bp, while K562-LenMatch contains length-matched origin and non-origin sequences( $< 512$ bp) to control for potential sequence-length bias. Metrics include accuracy, area under the ROC curve (AUC), precision, and recall

Performance on K562-L512, which retains the original sequence length distribution and is therefore the closest to the original K562 dataset, shows higher predictive performance than Ori-FinderH (AUC of 0.997 vs. 0.962, respectively). However, this dataset exhibits a substantial difference in length distributions between positive and negative sequences. In this setting, sequence length itself becomes a strong discriminative signal, meaning that the model can partially distinguish between the two classes based on length alone. Therefore, the observed improvement in performance likely reflects, at least in part, the DNABERT model exploiting this difference in sequence length. In contrast, the model fine-tuned on K562-LenMatch achieved an accuracy of 0.894 and an AUC of 0.956. Although this performance is lower than that observed on the K562-L512 dataset, it represents a more conservative and controlled evaluation. In K562-LenMatch, the length distribution of negative samples was adjusted to match that of the positive samples, thereby removing sequence length as a discriminative cue and forcing the model to rely on sequence composition rather than length differences. By eliminating this confounding factor, the K562-LenMatch dataset helps direct the model towards detecting sequence patterns underlying human replication origins. Therefore, for interpretability analyses, we focus on the model fine-tuned on the K562-LenMatch dataset.

The results for chromosome-based splitting are reported in Additional File 1, Table S6, as the average performance across the seven splits (mean and standard deviation) for both datasets. The comparable performance to that observed under random splitting suggests that the model does not rely on memorization of homologous sequences, but instead learns generalizable representations for this downstream task.

To investigate which sequence features contribute to the model’s predictions, we applied the attention-based explainability DNABERT pipeline described in section Attention-based explanation, for the K562-LenMatch dataset. Based on predictions on corresponding independent test set, 14,729 out of 16,031 origin sequences were correctly classified as origins. Attention maps corresponding to these true positive sequences were analyzed to identify regions contributing most strongly to the model’s decisions.

**Fig. 4:**
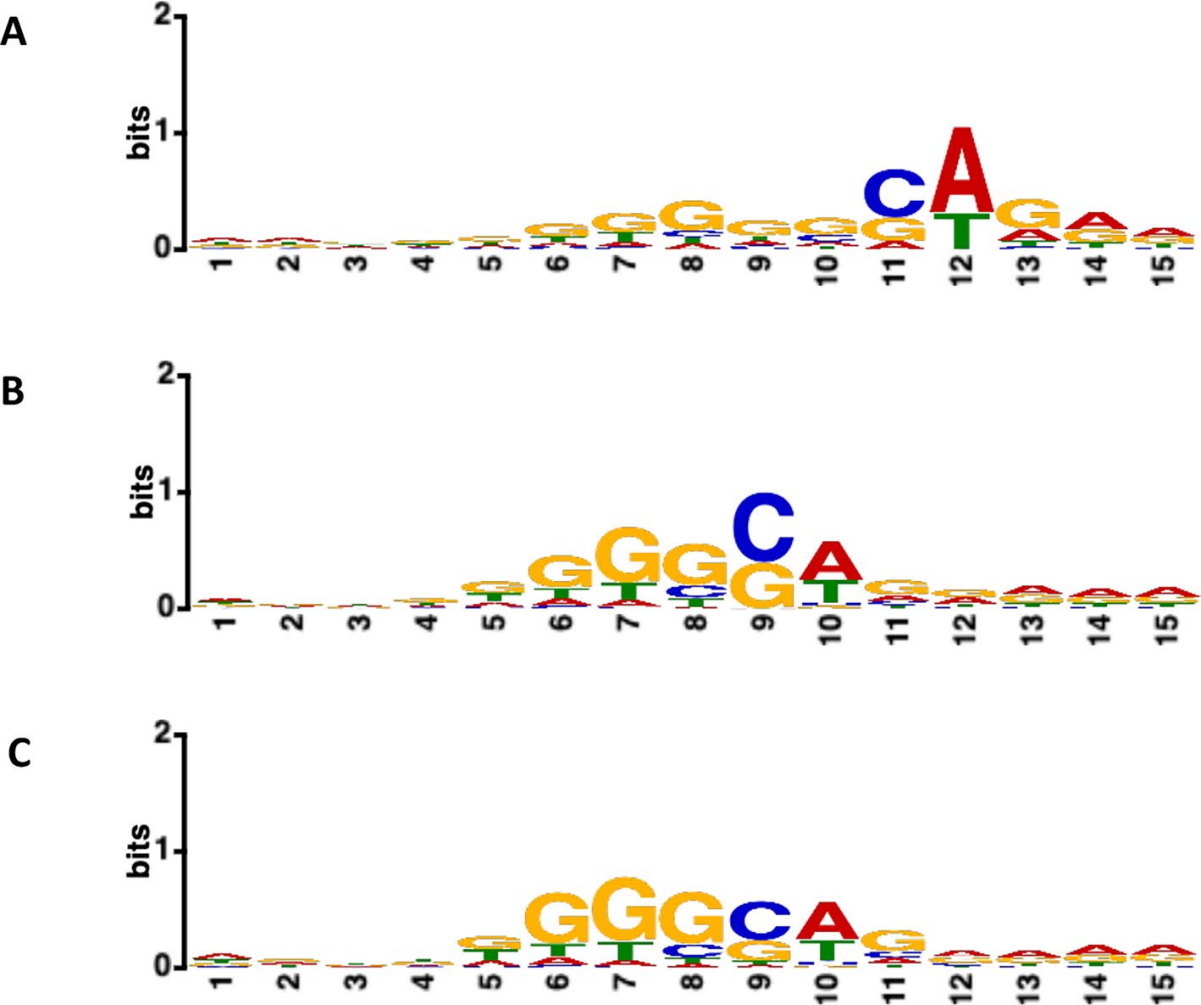
Motif discovery from high-attention fragments in true positive sequences of test set. Motifs were identified using MEME from 20 bp fragments surrounding attention peaks in sequences correctly classified by the DNABERT model fine-tuned on the K562-LenMatch dataset. **A** Motif discovered from fragments centered on the top three attention peaks (E-value = .0e–248). **B** Motif discovered from fragments centered on the top two attention peaks (E-value = 6.5e−295). **C** Motif discovered from fragments centered on the single highest attention peak (E-value = 1.2e−327)

Initially, we examined the three highest attention peaks, as in the yeast pipeline, within each sequence. To increase robustness of motif discovery, we extracted 20 bp fragments centered on attention peaks exceeding a threshold of 0.1 and analyzed them using MEME. Motif discovery identified a G-rich motif, as illustrated in Fig. 4 A.

To further refine the signal, we progressively restricted the search space by considering only fragments corresponding to the top two attention peaks, and finally only the single highest peak per sequence. The resulting motifs are shown in Fig. 4 B and Fig. 4 C. As the analysis focuses on increasingly strong attention signals, the discovered motifs display progressively stronger G enrichment within their core region.

The G-rich nature of the extracted motifs is consistent with previous observations that human replication origins frequently occur in GC-rich genomic regions and are often associated with G-quadruplex-forming sequences [42, 43]. Genome-wide analyses have also identified conserved G-rich sequence elements, including poly-G runs such as GGGG motifs, as informative features for predicting human replication origin activity, [44].

## Discussion

In this work, we tackled the challenge of predicting DNA replication origins with two transformer-based models, emphasizing data design and interpretability. We constructed biologically motivated datasets with different negative-sampling strategies for model fine-tuning and complemented them with controlled shuffled-sequence evaluation settings to probe model behavior under sequence perturbations. This design enabled us to investigate which sequence features were captured by the models and how these features influence predictions. To further explain the predictions, we developed tailored interpretability pipelines for each model by leveraging established bioinformatics and AI tools. Together, these analyses offer new insights into model behavior and highlight the biological signals that can be recognized by the models in replicating origin sequences.

Direct comparison with previously published ORI prediction methods is challenging because no standardized benchmark dataset exists for this task. Existing studies differ substantially in dataset construction, sequence length, preprocessing strategies, negative sampling procedures, and feature representations. For example, Wu et al. [22] proposed a deep learning framework combining Word2Vec-based sequence embeddings with a convolutional neural network trained from scratch. In this approach, DNA sequences are first segmented and converted into numerical vectors using Word2Vec before being used as inputs to the CNN classifier. The model reported high predictive accuracy for *S. cerevisiae* when evaluated using cross-validation on curated benchmark datasets. However, these datasets involve extensive preprocessing steps, including sequence segmentation and filtering of homologous samples, and therefore differ substantially from the datasets used in our study, making direct quantitative comparison difficult. Word2Vec-based representations also require explicit sequence preprocessing prior to model training.

To provide context for our results, we therefore focus on studies whose datasets are conceptually most similar to ours. Singh et al. [18] proposed a multi-view ensemble learning (MEL) framework to identify DNA replication origins in yeast. Their approach integrates multiple feature subsets derived from sequence composition as well as physical and structural DNA properties, with separate classifiers trained for each feature view and combined using a performance-weighted strategy. The method was evaluated on the nrACS dataset, which consists of 251 non-replicating ACS (nrACS) sequences extracted from genomic regions spanning −100 bp to +166 bp relative to the nrACS location, resulting in sequences of length 266 bp. This dataset is conceptually similar to our ACS-Neg dataset, although the sequence length is shorter. The best reported performance for the nrACS dataset is AUC = 0.76. Within this context, the AUC values obtained by DNABERT and DNABERT-2 on our ACS-Neg dataset (0.82 and 0.72, respectively) fall within a comparable range of predictive performance.

Another study by Do and Le [19] applied extreme gradient boosting (XGBoost) using hybrid sequence-derived descriptors to classify replication origin sequences. In that study, the dataset was constructed from experimentally confirmed ORIs obtained from OriDB, using 300 bp sequences with 405 ORIs as positive samples and 406 non-ORI sequences as negative samples randomly selected from the genome after excluding sequences similar to ORIs. This dataset is conceptually similar to our Random-Neg dataset. The XGBoost model reports an accuracy of 0.89 on this benchmark dataset. However, several methodological differences should be considered when interpreting this result. First, the sequences used in that study were shorter than those used in our dataset. Second, the negative samples were generated by excluding sequences with similarity to ORIs, which may simplify the classification task. In contrast, when constructing our dataset we did not explicitly remove negative samples with similarity to ORIs, which may better reflect realistic genomic conditions for this task.

Importantly, unlike these two and many previous approaches that rely on manually engineered features or embedding-based preprocessing pipelines derived from sequence composition or structural DNA properties, our method leverages pretrained genome language models that learn contextual sequence representations directly from raw DNA sequences. The goal of our study was therefore not to optimize predictive accuracy on a single benchmark dataset, but rather to investigate whether such models can provide robust interpretability while maintaining predictive performance within the range reported for existing approaches.

We showed that one of the key factors to distinguish ORIs in budding yeast is the presence of ACS motifs, particularly for DNABERT. Our motif discovery pipeline, which extracts motifs from high-attention fragments of sequences, highlights DNABERT’s ability to identify biologically meaningful sequence motifs, showing strong alignment with ACS patterns. While DNABERT-2 does not exhibit visually distinguishable high attention scores for ACS motifs, our perturbation-based explainability analysis confirms that eliminating these motifs impacts its predictions, suggesting that ACS motifs, alongside other features, contribute to the decision-making process of this model.

Our analysis revealed that tokenization plays a critical role in shaping how sequence features are represented and processed by the models. By comparing the attention heatmaps of both models, it can be concluded that DNABERT has the capacity to integrate global sequence dependencies beyond just local context. Moreover, insights from perturbation-based explanations suggest that DNABERT-2’s BPE tokenization strategy biases the model toward short-range patterns by weighting individual tokens more strongly. While DNABERT learns both the token identity and the larger sequence context, DNABERT-2 relies more heavily on token identity, when strong origin-associated sequence signals are present, although sequence ordering can still influence predictions.

Using our SHAP-based explainability pipeline for DNABERT-2, we found that the tokens receiving the highest SHAP attribution scores in origin prediction were predominantly A/T-rich. Because SHAP estimates feature contributions under a perturbation-based approximation, these attributions reflect how the model assigns importance to input tokens rather than definitive causal determinants of model behavior. Nevertheless, the consistent enrichment of A/T-rich tokens is compatible with the known A/T-rich sequence composition of yeast replication origins. Importantly, this observation aligns with the signals identified through our complementary analyses, including attention-based motif recovery and perturbation experiments.

Importantly, we do not interpret the observed performance differences as evidence of statistical superiority of one model over the other. Rather, the comparison is intended to highlight differences in model behavior across datasets and to examine how the respective tokenization strategies influence interpretability and sequence feature representation.

To assess whether the proposed interpretability framework generalizes beyond yeast, we applied the DNABERT pipeline to human replication origin sequences using the K562 dataset. Unlike budding yeast, where replication origins are defined by the well-characterized ARS consensus sequence (ACS), replication origins in higher eukaryotes lack a strict consensus motif and are strongly influenced by chromatin context and transcriptional regulation [45, 46]. Consistent with previous studies reporting enrichment of G-quadruplex—forming sequences near human replication origins [42, 43], our attention-based motif discovery identified G-rich sequence patterns associated with correctly predicted origin sequences. Although this sequence signal is weaker than the ACS motif observed in yeast, it indicates that DNABERT can still capture biologically meaningful sequence features linked to replication initiation. This finding is consistent with the notion that sequence determinants of human replication origins are more heterogeneous than in yeast. Clustering of attention-derived sequence fragments may therefore help reveal distinct subclasses of sequence patterns contributing to origin activity.

## Conclusions

This study investigated the effectiveness of transformer-based language models in predicting DNA replication origins in *S. cerevisiae* genome and examined the sequence properties they capture. Using a series of datasets constructed through controlled perturbations of negative samples, we systematically evaluated how pretrained genomic language models represent known origin-associated sequence signals.

In evaluating DNABERT and DNABERT-2, we found that both models can be effective, albeit in distinct ways, primarily due to differences in their tokenization strategies. Our analysis shows that tokenization influences how models represent ordered sequence dependencies and affects the interpretability of recovered sequence patterns.

To further probe these differences, we developed customized interpretability pipelines for each model. An attention-guided motif discovery pipeline applied to DNABERT successfully recovered biologically meaningful motifs, including the ACS pattern, in a system where the sequence determinants of replication origins are well characterized, thereby providing a validation framework for interpretability analyses. For DNABERT-2, we complemented attention-based analyses with perturbation-based approaches, including an adaptation of the TransSHAP framework, enabling attribution-based interpretation of the tokens contributing to model predictions.

These findings suggest that tokenization should be considered not only from the perspective of predictive performance but also of biological interpretability. Future genomic foundation models may therefore benefit from biology-aware tokenization strategies that incorporate prior biological knowledge, such as sequence motifs or regulatory elements, while preserving the flexibility of data-driven approaches. Whether such task-specific tokenization improves interpretability, predictive performance, or both remains an important direction for future research.

Although our study focuses on *S. cerevisiae*, where the well-defined ACS motif provides a benchmark for motif analysis, the fundamental principles of DNA replication initiation are highly conserved across eukaryotes [38]. Accordingly, the insights gained from yeast provide a foundation for applying this interpretability framework to more complex genomes, as illustrated by an exploratory analysis of human replication origin sequences.

## Supplementary material

Below is the link to the electronic supplementary material.

Supplementary file 1 (PDF 5 kb)

Supplementary file 2 (CSV 5 kb)

Supplementary file 3 (CSV 5 kb)

Supplementary file 4 (CSV 5 kb)

Supplementary file 5 (CSV 5 kb)

## Data Availability

All datasets for the budding yeast genome used in this study are available in the Supplementary Information. They were generated from the data set originally proposed by OriDB for the origins of DNA replication in the budding yeast genome which is publicly available on the corresponding OriDB website: https://oridb.org. All codes and datasets (for both the budding yeast and the human genome) supporting our explainability pipelines are available in the corresponding GitHub repository: https://github.com/Piroozeh/DNAOrigin Prediction.

## Abbreviations

ORIs: Replication origins
ARS: Autonomous replication sequence
ACS: ARS consensus sequence
ORC: Origin recognition complex
SVM: Support vector machines
MEL: Multi-view ensemble learning
CNN: Convolutional neural network
LLMs: Large language models
TP: True positive
BPE: Byte pair encoding
MEME: Multiple expectation maximization for Motif elicitation
NLP: Natural language processing
SHAP: Shapley additive explanations
SD: Standard deviation
AUC: Area under the curve

## Footnotes

1 Here, ‘W’ denotes A or T, ‘Y’ denotes C or T, and ‘R’ denotes G or A.
