# Supplementary material for: Interpretable prediction of DNA replication origins in S. cerevisiae using DNABERT and DNABERT-2
Source: BMC Bioinformatics. 2026 Jul 22;27:157. doi: 10.1186/s12859-026-06562-5 (PMC13393499; doi:10.1186/s12859-026-06562-5)
Supplement: Supplementary file 1 — (PDF 5 kb) [file 12859_2026_6562_MOESM1_ESM.pdf]

Additional File 1: Supplementary Figures and Tables

Supplementary information: Interpretable prediction of DNA replication origins in *S. cerevisiae* using DNABERT and DNABERT-2;

Zohreh Piroozeh, Ildem Akerman, Olga V.Kalinina, Stefan Kesselheim, Alina Bazarova

1. SUPPLEMENTARY FIGURES

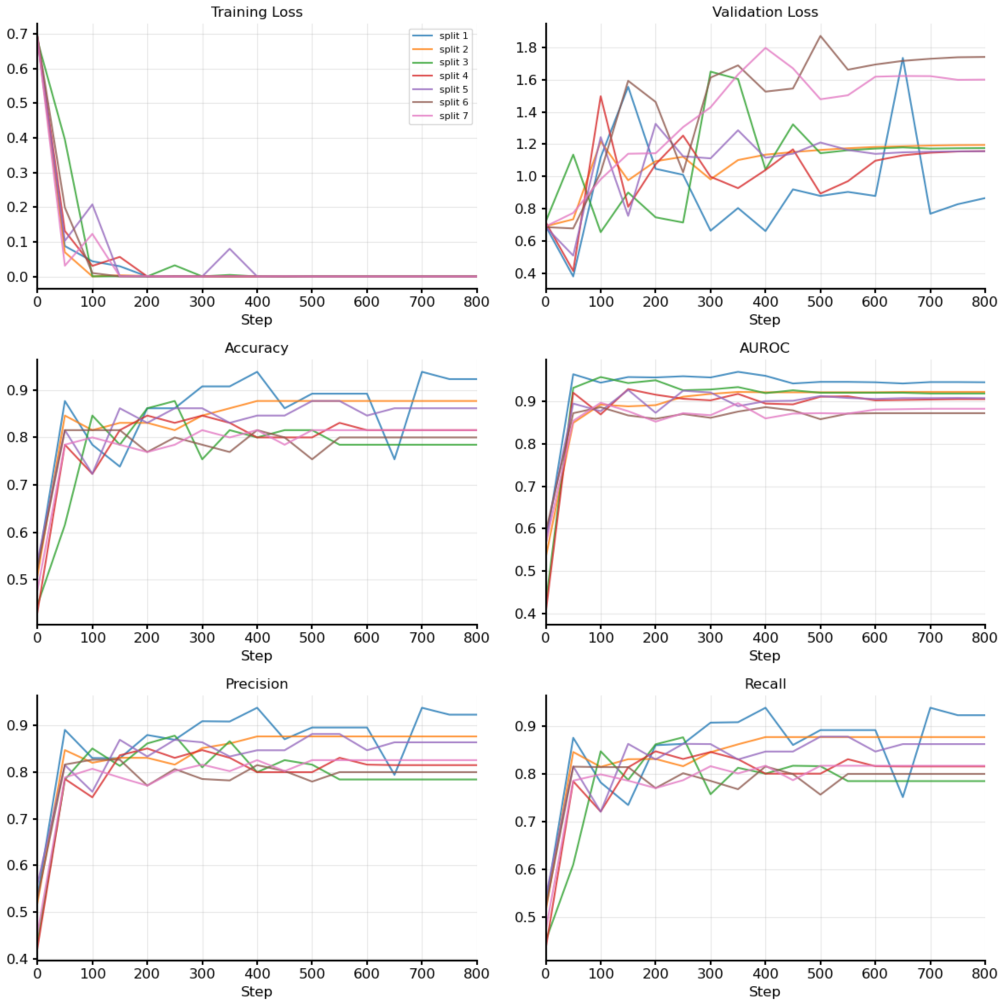

Fig. S1. Training and validation loss together with evaluation metrics for DNABERT on the Random-Neg dataset. The model was trained for 200 epochs (800 steps). Curves correspond to the seven independent train/validation/test splits used in the study. We report training and validation loss (top row). Metrics shown include validation accuracy, AUROC, precision, and recall. The gradual increase in validation loss despite stable evaluation metrics likely reflects the limited dataset size and the sensitivity of cross-entropy loss to a small number of misclassified validation samples during later training stages, see section A for more details.

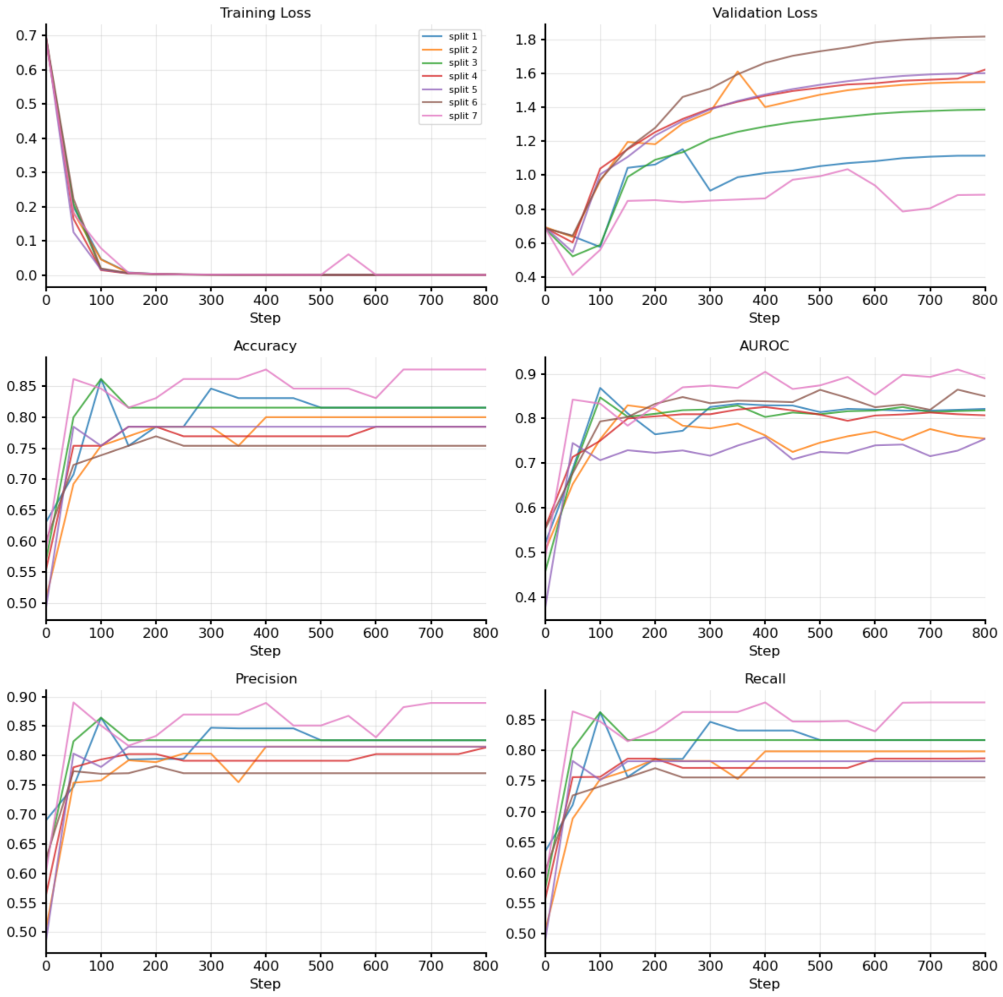

Fig. S2. Training and validation loss together with evaluation metrics for DNABERT-2 on the Random-Neg dataset. The model was trained for 200 epochs (800 steps). Curves correspond to the seven independent train/validation/test splits. We report training and validation loss (top row). Metrics shown include validation accuracy, AUROC, precision, and recall. The gradual increase in validation loss despite stable evaluation metrics likely reflects the limited dataset size and the sensitivity of cross-entropy loss to a small number of misclassified validation samples during later training stages.

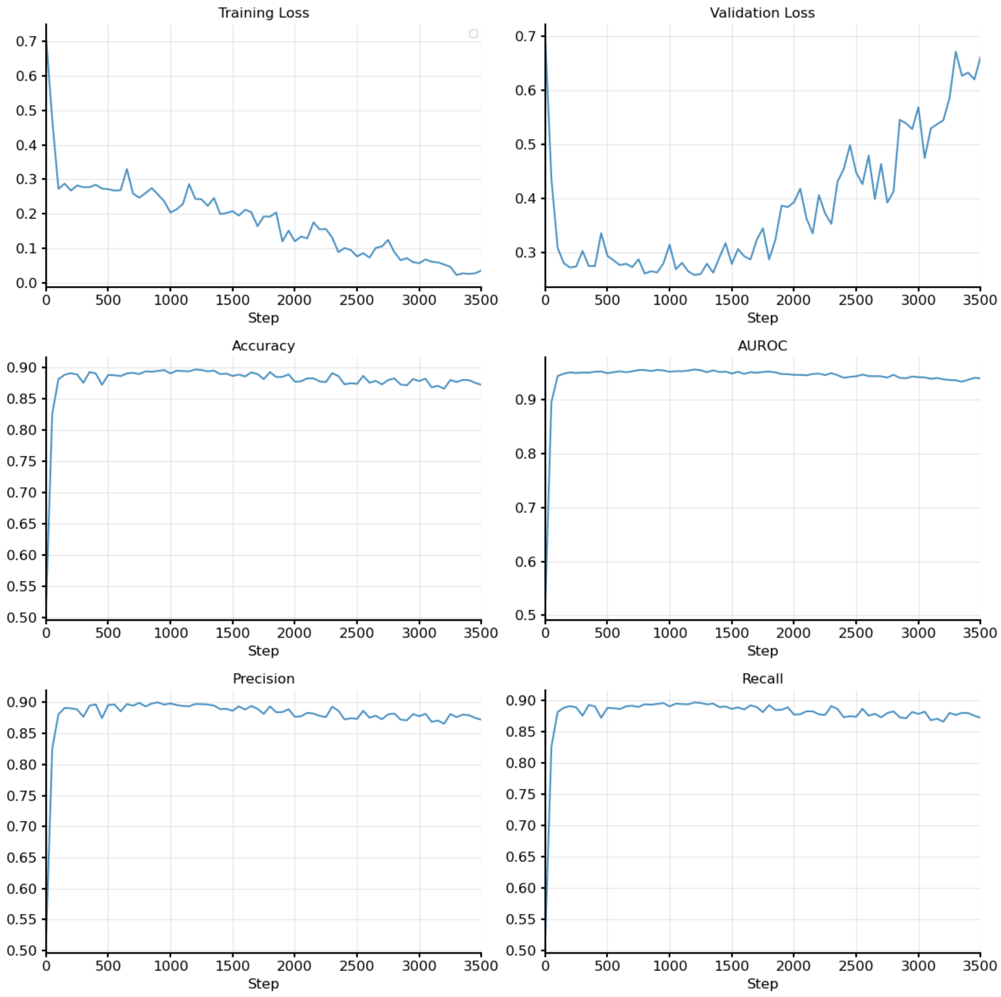

Fig. S3. Training and validation loss together with evaluation metrics for DNABERT on the modified human K562-LenMatch dataset. The model was trained for 7 epochs (3500 steps). We report training and validation loss (top row). Metrics shown include validation accuracy, AUROC, precision, and recall

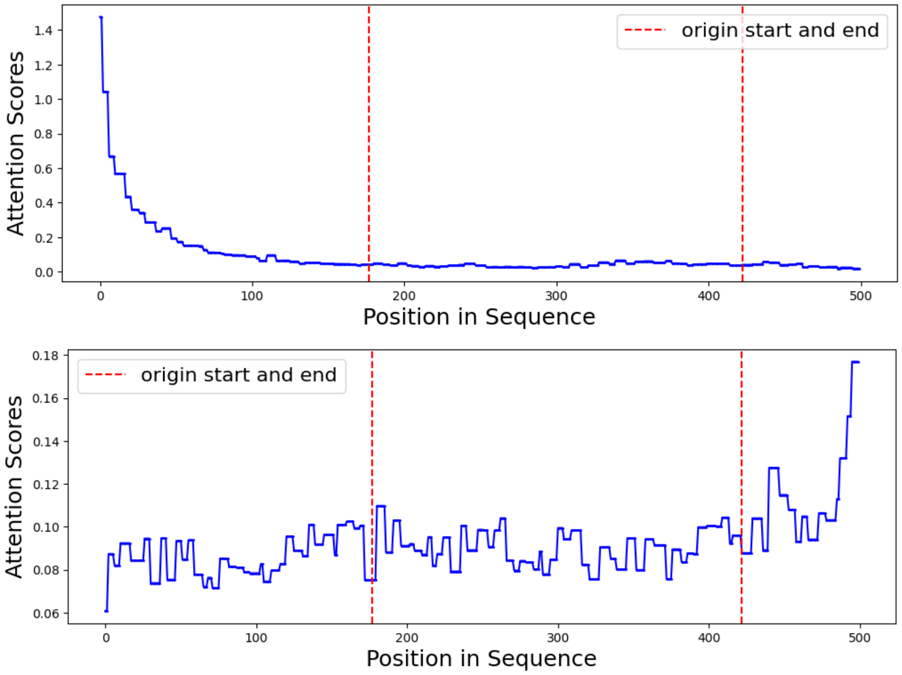

A

B

Fig. S4. Attention scores extracted from the final layer of the fine-tuned DNABERT-2 model. Attention scores are shown for a representative sequence. Red dashed lines indicate annotated origin start and end positions. (A) Attention scores corresponding to the [CLS] token, which serves as a global representation of the input sequence. (B) Alternative visualization showing average attention scores across all tokens. Neither representation reveals clearly localized high-attention regions.

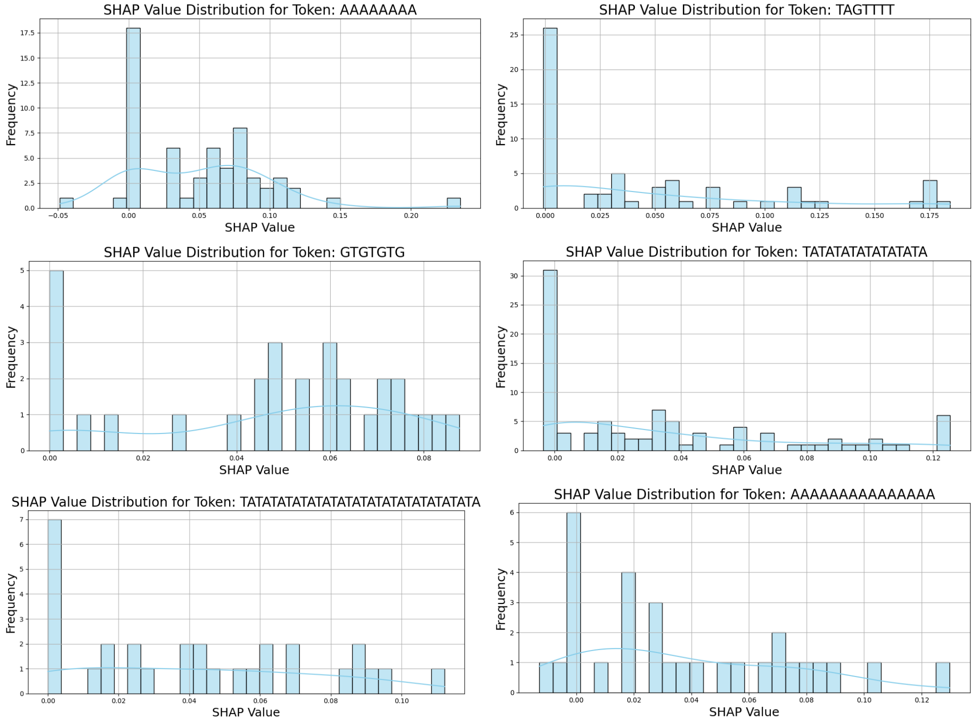

Fig. S5. Distribution of SHAP values for some of the top tokens listed in Table 4, for DNABERT-2 predictions. Distributions are skewed toward positive values, indicating that these tokens consistently contribute to classifying sequences as origins. A considerable number of zero values are also observed, which result from SHAP's built-in L_1 regularization that suppresses weak attributions. Overall, these patterns demonstrate that these tokens recur as important in DNABERT-2 predictions, since they consistently exhibit strong positive predictive power in distinguishing origin sequences, across multiple runs of SHAP analysis.

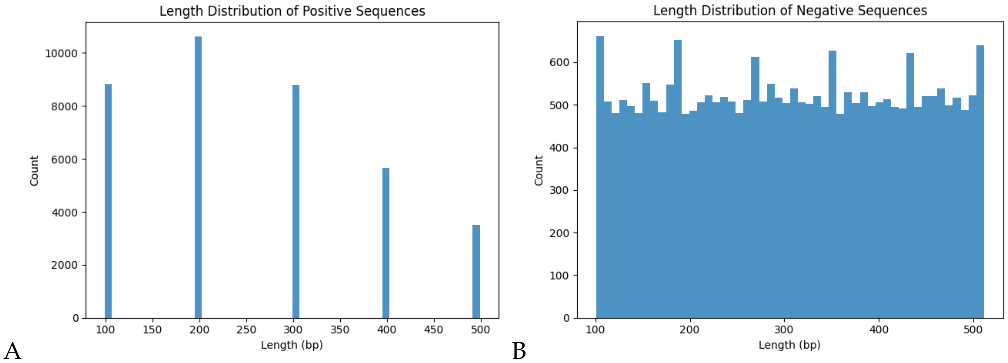

Fig. S6. Distribution of sequence length in dataset K562-L512 (sequences shorter than 512), A: length distribution of positive (origin) sequences, B: length distribution of negative (non-origin) sequences.

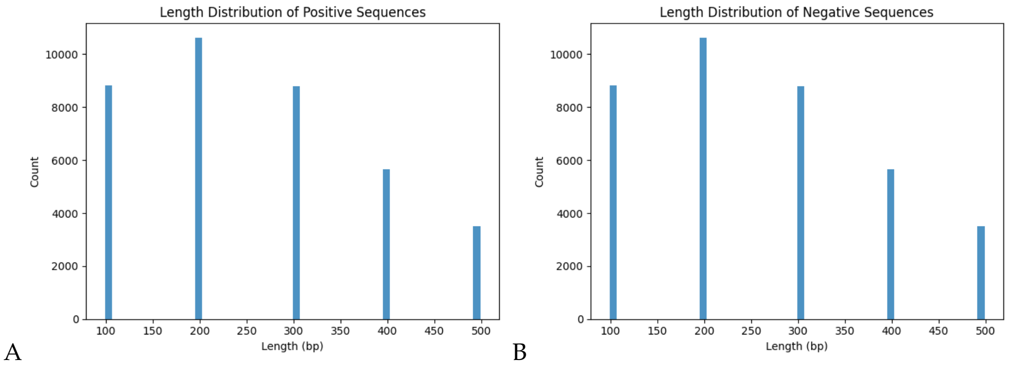

Fig. S7. Distribution of sequence length in dataset K562-LenMatch, A: length distribution for positive (origin) sequences, B: length distribution for negative (non-origin) sequences.

2. SUPPLEMENTARY TABLES

Table S1. Hyperparameter settings used for fine-tuning DNABERT across all datasets.

Model	learning rate	warmup	batch size
DNABERT (Budding yeast datasets)	2×10^{-4}	0.1 (percent)	32
DNABERT-2 (Budding yeast datasets)	3×10^{-5}	50 (steps)	32
DNABERT (Human genome datasets)	2×10^{-4}	0.1 (percent)	32

Table S2. Average validation performance of DNABERT across the four datasets. Values represent the mean metrics across seven runs; standard deviations are shown in parentheses.

Dataset	Accuracy	AUC	Precision	Recall
Random-Neg	0.86 (0.04)	0.92 (0.03)	0.87 (0.04)	0.86 (0.04)
ACS-Neg	0.76 (0.04)	0.80 (0.04)	0.77 (0.04)	0.76 (0.04)

Table S3. Average validation performance of DNABERT-2 across the four datasets.. Notation as in Table S2

Dataset	Accuracy	AUC	Precision	Recall
Random-Neg	0.82 (0.05)	0.85 (0.04)	0.83 (0.04)	0.82 (0.05)
ACS-Neg	0.74 (0.05)	0.74 (0.05)	0.75 (0.04)	0.74 (0.05)

Table S4. Average test performance of DNABERT and DNABERT-2 using chromosome-based data splitting on the Random-Neg dataset. Values represent the mean across seven independent splits with standard deviation in parentheses.

Model	Accuracy	AUC	Precision	Recall
DNABERT	0.85 (0.03)	0.91 (0.03)	0.85 (0.03)	0.85 (0.03)
DNABERT-2	0.82 (0.05)	0.83 (0.04)	0.84 (0.03)	0.82 (0.05)

Table S5. Distribution of positive (origin) and negative (non-origin) samples across training, validation, and test sets for human origin datasets, in random splitting.

	Train set		Validation set		Test set	
Dataset	Positive	Negative	Positive	Negative	Positive	Negative
K562-L512	29950	20938	7488	5235	16031	11284
K562-LenMatch	29950	29950	7488	7488	16031	16031

Table S6. Average test performance of DNABERT using chromosome-based data splitting on two K562 datasets with different length distributions. K562-L512 corresponds to the dataset containing sequences shorter than 512 bp, while K562-LenMatch contains length-matched origin and non-origin sequences($<$ 512 bp) to control for potential sequence-length bias. Values represent the mean across seven independent splits with standard deviation in parentheses.

Dataset	Accuracy	AUC	Precision	Recall
K562-L512	0.995 (0.002)	0.998 (0.003)	0.995 (0.002)	0.994 (0.003)
K562-LenMatch	0.892 (0.011)	0.954 (0.007)	0.892 (0.014)	0.891 (0.009)

Table S7. Tokens contributing to non-origin predictions by DNABERT-2 based on SHAP analysis. "Frq. in 30 runs" indicates the number of SHAP runs (out of 30) in which the token appeared among the top contributors. "Total Frq." represents the cumulative number of occurrences across runs, and "Avg. SHAP values" denotes the mean Shapley value across these occurrences.

Token	Frq. in 30 runs	Total Frq.	Avg. SHAP values
TCACCCA	13	13	0.104691
GTACAGG	9	9	0.095725
CCGTTA	9	14	0.088946
TCAGG	8	12	0.079529
CCTTTG	7	8	0.117304
GAAGTAA	7	7	0.115140
TCGCTT	7	7	0.088657
CTTCTA	7	8	0.085571
TTACC	7	13	0.083307
GGGTGA	7	7	0.083294
CAGGTT	7	11	0.079946
GCGA	6	8	0.135927
TCATCTT	6	6	0.107194
GCCTT	6	20	0.097800
TACCA	6	8	0.086110
TCTTTT	6	10	0.079969
CCAGG	6	8	0.078441
GCAGTCC	5	5	0.125671
GGTGAAA	5	6	0.120022
GGAGG	5	9	0.119330
TAACC	5	7	0.106951
GTAA	5	19	0.099786
GTCTT	5	15	0.098598
GGCC	5	12	0.097743
TCGGTA	5	7	0.097103
CCATTTT	5	5	0.089352
CATTA	5	6	0.086596
TCGTAA	5	6	0.086230
CCCGG	5	5	0.086158
GGCTA	5	6	0.084083
TCCCTTA	5	5	0.082238
TTAGG	5	7	0.079303
CGTCTG	5	5	0.077822

Table S8. SHAP values for true negatives (TN) that remained correctly classified after token shuffling. Positive SHAP values (right columns) contribute toward classifying instances as negative, while negative SHAP values (left columns) contribute toward classification as positive.

Token	Frq. In 30 runs	Total Frq.	Avg. SHAP values	Token	Frq. In 30 runs	Total Frq.	Avg. SHAP values
GAAAA	16	16	-0.0501265	CGTT	13	20	0.07171644
TAAATAAAA	16	16	-0.0915546	CCTGTT	12	12	0.09165467
TATAAA	12	12	-0.0669559	CCTCTG	12	12	0.09154788
GAGA	11	13	-0.0436362	CGG	11	55	0.06555112
CATAA	10	10	-0.0404049	TTCA	11	15	0.06215885
TAAATA	10	10	-0.053998	CCTT	10	15	0.08676739
GTGTATG	9	9	-0.053498	GAAGG	10	11	0.08272548
CTGTG	9	9	-0.0579393	GATAA	10	16	0.06975239
GTGTG	8	9	-0.044732	CTTTCC	9	9	0.08515323
TAAACAA	8	8	-0.0522588	TCACC	9	11	0.07267863
TCTT	7	8	-0.0292618	TGATGA	9	10	0.06998401
TGCA	7	7	-0.0385677	CGA	8	13	0.08799232
TAAGTG	7	7	-0.0475368	CCTGGTG	8	8	0.07912081
GCATGCA	7	7	-0.0494099	CCATT	8	14	0.07468361
TAATG	7	7	-0.052489	TCTCTT	8	10	0.07272883
TATT	6	6	-0.0344339	CCTG	8	12	0.07237199
CAAAA	6	8	-0.0379254	CTCC	8	14	0.07063834
TGTTTGTT	6	6	-0.0389024	TCACAGG	8	8	0.06223685
TGTT	6	6	-0.0454248	TCCTT	7	10	0.09847074
CAATAAA	6	6	-0.0489026	CTTTT	7	9	0.09506912
AAAAAA	6	6	-0.05579	CAACGG	7	7	0.08636836
GG	5	5	-0.0150232	TCGGTG	7	7	0.07815149
CGA	5	5	-0.0203779	GATGA	7	11	0.07694274
GTA	5	5	-0.0208271	GAATT	7	9	0.07559819
CAATG	5	5	-0.0223627	CTTCCA	7	8	0.07556828
CCA	5	7	-0.0227003	TCGCC	7	7	0.07294573
GAATAA	5	5	-0.0302639	CTAA	7	8	0.06981752
CCAA	5	5	-0.0310721	CAGGTT	7	7	0.06921968
GAGAA	5	5	-0.0359836	TCAA	7	20	0.06445167
GAGTG	5	5	-0.0391031	GCTAA	7	7	0.06170417
CTTTTTT	5	5	-0.0435984	GTCC	7	10	0.06055339
GGAGG	5	5	-0.0453685	GCGG	7	11	0.05935779
TTTA	5	6	-0.0463321	TCGC	6	7	0.10306828
TGAA	5	5	-0.0521597	CATCA	6	9	0.09015714
CAAATG	5	5	-0.0523583	TCTTCTT	6	9	0.08817028
TGATTA	5	5	-0.0602409	TCAGG	6	6	0.08643605

Table S9. SHAP values for true negatives that became false positives after token shuffling. Positive SHAP values (left columns) contribute toward classification as positive, whereas negative SHAP values (right columns) contribute toward classification as negative.

Token	Frq. In 30 runs	Total Frq.	Avg. SHAP values	Token	Frq. In 30 runs	Total Frq.	Avg. SHAP values
TATA	23	42	0.03875838	CGG	28	60	-0.0389651
CACACACA	21	27	0.03956291	TCGGTA	23	32	-0.0425716
TATTTG	18	21	0.04429985	GCTCAA	21	21	-0.0572318
TTTTTTTT	16	16	0.03933014	CCTTA	21	26	-0.0446305
TAAA	16	23	0.04340432	CCTT	17	20	-0.0292082
TAATGTG	15	15	0.05733218	GGTT	16	16	-0.0349263
TCTA	14	15	0.03450688	GAA	16	20	-0.0296719
TGTG	14	21	0.04277304	GCC	16	19	-0.0277371
TATTTT	14	16	0.04774167	GATG	15	18	-0.0419336
GTG	12	13	0.02763339	GCTATTA	15	15	-0.0366447
TCTAA	12	12	0.03523927	GCGATA	14	14	-0.0359096
TATATG	12	14	0.03812827	CCCTTAA	14	14	-0.0311289
TGAA	12	14	0.03833912	GGCA	14	16	-0.0216312
TGATTAAA	12	13	0.04213458	GGGAA	13	13	-0.0401361
GCCAA	11	11	0.02406089	CCTCA	12	12	-0.0323772
TGATTA	11	11	0.03978886	GGAA	12	12	-0.032112
CGC	10	10	0.02573203	CAAAA	11	12	-0.0196575
CTATATA	10	11	0.03162189	CAGGA	10	10	-0.0579583
TACA	10	11	0.03178498	CGTT	10	10	-0.0390793
TGTA	10	10	0.04132797	GGTG	10	12	-0.0329277
TTTA	9	9	0.03190549	GCCA	9	9	-0.041559
CTTTTAA	9	9	0.03569259	CCTAA	9	10	-0.0390766
GTGTG	9	9	0.04156017	GTT	9	9	-0.0355233
CGAGG	9	10	0.04479859	CGACC	9	9	-0.0338793
TGTTTG	9	10	0.04854075	GGAAA	8	8	-0.0435814
GTCC	9	9	0.0529122	GCGGCC	8	8	-0.041509
CAAAA	9	9	0.05380813	GAATT	8	8	-0.0365952
GTTGAA	9	9	0.06713737	GAATTTG	8	8	-0.0343026
GATT	8	8	0.02246176	GCCTT	8	8	-0.0326573
CTAA	8	8	0.03213073	CCATGGA	8	8	-0.0322482
TAACAA	8	9	0.03601745	GGTGA	8	8	-0.0304464
TAGGAAAA	8	8	0.0395264	CCCAGAA	8	8	-0.0255667
TAAAA	8	9	0.04883378	GCGATG	7	7	-0.0606199
TATATT	8	8	0.06790283	GTCCATT	7	7	-0.0362559
CATTAAA	7	7	0.03355504	CCAATT	7	7	-0.0351483
GCCAGG	7	7	0.03762948	GAAGG	7	7	-0.0321603
GTGCC	7	7	0.04152102	CAAGG	7	7	-0.0275919

Table S10. AT-richness index across sequence groups and statistical comparisons. The table reports group size (n), mean and median AT-index values, Mann–Whitney U test p-values, and Cliff's δ effect sizes for comparisons between sequence groups.

Group	n	Mean AT-index	Median AT-index	Comparison	p-value (MWU)	Cliff's $\|\delta\|$	Effect size
TP_original	61	0.609	0.504	TP vs TN	3.39×10^{-14}	0.870	large
TN_original	44	-0.448	-0.405	TP vs TN→FP	2.28×10^{-5}	0.731	large
TN→FP	14	-0.173	-0.212	TN vs TN→FP	0.14	0.265	small
TN→TN	30	-0.500	-0.499	TN vs TN→TN	0.54	0.085	negligible

3. SUPPLEMENTARY TEXT

A. Loss behaviour

Validation binary cross-entropy (BCE) loss and discrimination metrics such as accuracy and AUROC quantify different aspects of model behaviour. The BCE loss is defined as

$$\mathrm{BCE} = -\frac{1}{N}\sum_{i=1}^{N}[y_i \log(p_i) + (1 - y_i)\log(1 - p_i)], \quad \text{(S1)}$$

where p_i denotes the predicted probability for sample i, $y_i \in 0, 1$ is the corresponding true class label, and N is the total number of samples.

Unlike accuracy and AUROC, which primarily measure the model's ability to discriminate between classes, BCE is additionally sensitive to prediction confidence. Because the logarithmic penalty increases sharply for highly confident incorrect predictions (e.g., ($p_i \to 1$) when $y_i = 0$), even a small number of increasingly confident misclassifications can contribute disproportionately to the overall loss. Consequently, validation BCE loss may increase during training despite relatively stable, or even improving, discrimination performance as measured by accuracy and AUROC.

B. AT-index calculation

To quantify origin-like sequence composition independently of SHAP attribution scores, we computed several interpretable sequence descriptors capturing AT-rich features commonly associated with yeast replication origins. For each sequence we calculated the following quantities:

1. **AT fraction**, defined as the proportion of nucleotides that are adenine or thymine.
2. **Motif count**, representing the number of occurrences of AT-rich motifs identified from the SHAP analysis of true positive predictions.
3. **Longest AT run**, defined as the length of the longest contiguous stretch of A/T nucleotides.
4. **Alternating AT count**, representing the number of alternating A/T patterns (e.g. TATA-like tracts).

To combine these descriptors into a single measure of origin-like sequence signal, each feature x_j was standardized across all analyzed sequences:

$$z_{ij} = \frac{x_{ij} - \mu_j}{\sigma_j},$$

where μ_j and σ_j denote the mean and standard deviation of feature j across all sequences.

The *AT-richness index* for sequence i was then defined as the unweighted average of the four standardized descriptors:

$$AT\_index_i = \frac{1}{4}\sum_{j=1}^{4} z_{ij}.$$

This index provides a normalized measure of local AT-rich sequence signal.

The AT-index was computed for four sequence groups: true positives (TP_original), true negatives (TN_original), negatives that remained correctly classified after token shuffling (TN→TN), and negatives that became false positives after token shuffling (TN→FP).

Group differences were evaluated using the Mann–Whitney U test, and Cliff's δ was calculated as a non-parametric effect size measure. The latter quantifies the probability that a randomly selected value from one group is larger than a randomly selected value from another group, minus the reverse probability. It is defined as

$$\delta = \frac{\#(x_i > y_j) - \#(x_i < y_j)}{n_x n_y},$$

where x and y denote the two groups being compared and n_x, n_y are their respective sample sizes. The value of δ ranges from -1 to 1, where values close to zero indicate strong overlap between distributions. Following commonly used thresholds, effect sizes were interpreted as negligible ($|\delta| < 0.147$), small ($|\delta| < 0.33$), medium ($|\delta| < 0.474$), and large ($|\delta| \geq 0.474$).

Table S10 summarizes the AT-richness index across the analyzed sequence groups together with statistical comparisons. True positive sequences (TP_original, $n = 61$) exhibited substantially higher AT-index values than true negatives (TN_original, $n = 44$), with a highly significant difference (Mann–Whitney test $p = 3.39 \times 10^{-14}$) and a large effect size ($|\delta| = 0.87$). Similarly, TP sequences differed strongly from negatives that became false positives after token shuffling (TN→FP, $n = 14$; $p = 2.28 \times 10^{-5}$, $|\delta| = 0.73$). In contrast, the comparison between TN and TN→FP groups showed only a small effect size ($|\delta| = 0.27$) and did not reach statistical significance ($p = 0.14$), likely reflecting the relatively small sample size of the TN→FP subset ($n = 14$). Negatives that remained correctly classified after shuffling (TN→TN, $n = 30$) showed AT-index values comparable to the original negative set ($p = 0.54$, $|\delta| = 0.085$), indicating negligible differences between these groups.For reporting purposes, the absolute value $|\delta|$ is presented in Table S10, while the direction of the effect can be inferred from group medians.
